# A wearable, rapidly manufacturable, stability-enhancing microneedle patch for closed-loop diabetes management

**DOI:** 10.1038/s41378-024-00663-y

**Published:** 2024-08-19

**Authors:** Yiqun Liu, Li Yang, Yue Cui

**Affiliations:** 1 School of Materials Science and Engineering, Peking University, Beijing, 100871 China; 2 Renal Division, Peking University First Hospital, Peking University Institute of Nephrology, Key Laboratory of Renal Disease, Ministry of Health of China, Key Laboratory of Chronic Kidney Disease Prevention and Treatment (Peking University), Ministry of Education, Beijing, 100034 China

**Keywords:** Electrical and electronic engineering, Electronic properties and materials

## Abstract

The development of a wearable, easy-to-fabricate, and stable intelligent minisystem is highly desired for the closed-loop management of diabetes. Conventional systems always suffer from large size, high cost, low stability, or complex fabrication. Here, we show for the first time a wearable, rapidly manufacturable, stability-enhancing microneedle patch for diabetes management. The patch consists of a graphene composite ink-printed sensor on hollow microneedles, a polyethylene glycol (PEG)-functionalized electroosmotic micropump integrated with the microneedles, and a printed circuit board for precise and intelligent control of the sensor and pump to detect interstitial glucose and deliver insulin through the hollow channels. Via synthesizing and printing the graphene composite ink, the sensor fabrication process is fast and the sensing electrodes are stable. The PEG functionalization enables the micropump a significantly higher stability in delivering insulin, extending its lifetime from days to weeks. The patch successfully demonstrated excellent blood glucose control in diabetic rats. This work may introduce a new paradigm for building new closed-loop systems and shows great promise for widespread use in patients with diabetes.

## Introduction

Diabetes mellitus is a metabolic illness with a high level of blood glucose, and diabetes can cause numerous consequences, including heart disease, renal disease, retinopathy, and neuropathy^1,2^. The prevalence of diabetes has been in a large increase in virtually all regions of the world, and there are more than 415 million diabetic patients worldwide^3^. Closed-loop insulin infusion systems can provide an ideal option for managing and treating diabetes. It can calculate the insulin infusion doses according to blood glucose level, achieve an automated glucose management to obtain the near normal glycemic control in diabetic patients, and reduce the burden of frequent capillary blood glucose testing and insulin injections^4–6^. These commercial systems are usually composed of a subcutaneous biosensor for interstitial glucose, a subcutaneous pump for delivering insulin and an algorithm that automatically responds to changes in biosensor glucose levels and automatically adjusts insulin infusion^7^. However, the drawbacks of the current systems are too expensive for users to afford and too large, limiting their widespread use among diabetic patients.

Compared to the long needle biosensor for detecting subcutaneous interstitial glucose, microneedle biosensing devices are minimally-invasive, painless, miniaturized, portable and relatively safe^8–12^. Microneedle-based devices have been shown to enable up to 72 hours of continuous accurate glucose monitoring in human skin^13^ and up to 30 hours of continuous in vivo drug delivery^14^. An effective subcutaneous device or closed-loop device should be able to respond to the real-time blood glucose fluctuations, the sensing electrodes are essential components to achieve this goal. Recently, several microneedle biosensing devices and closed-loop devices have been fabricated based on different types of sensing electrodes.

For example, thin-film gold (Au) and silver (Ag) electrodes were fabricated on the sidewalls of 3D-printed microneedles based on the physical vapor deposition (PVD) technique, and the Ag electrode was further immersed in a ferric chloride (FeCl_3_) solution to form a silver/silver chloride (Ag/AgCl) layer for glucose sensing^15^. Au-multiwall carbon nanotubes and methylene blue were electrodeposited on microneedles as the sensing electrode and a quarter of the microneedles were metalized with silver as the reference electrode^16^. Au layer was electrodeposited on the microneedle array as the working electrode and Ag/AgCl layer was fabricated as the reference electrode^17^. Chromium (Cr) and Au layers were deposited on the microneedles as the working electrode and a quarter of the microneedles were evaporated with Ag and chlorinated with sodium hypochlorite to form the reference electrode (Ag/AgCl)^18^. A silicon nanowire field-effect transistor array was fabricated on microneedles for glucose sensing^19^. Pt and Ag/AgCl wires^20^ or metalized-carbon paste^21^ were integrated within the aperture of hollow microneedles to be as sensing electrodes. A closed-loop patch was constructed based on the chitosan microneedle arrays with sputtered thin-film Au and Ag electrodes, and the Ag electrode was further chlorinated by immersion in FeCl_3_, which turns into Ag/AgCl^22^. However, these methods have complex procedures to fabricate the electrodes and are expensive.

Graphene is an appealing nanomaterial with high surface area, mechanical strength, electrical conductivity, and biocompatibility^23,24^. Further, it can be simply combined with other materials to form composite inks. Graphene composite ink promises to be an excellent choice for making sensing electrodes, due to its simplicity and low cost.

The pumping device in the closed-loop system has a significant effect on the overall cost and size of the entire system. A few pumps have been studied for delivering insulin, such as the commercial mechanical pump driven by the electro-motor^25^, electroosmotic pump^26^, micro-gear pump^27^, diffusion-based micropump^28^, piezoelectric actuated insulin pump^29^, and electro-active polymer actuated insulin pump^30^. Among them, an electroosmotic pump is based on promoting the movement of mass fluid through the capillary or porous membrane with an electric field^31,32^, which is an appealing approach for delivering insulin. We have recently shown the delivery of insulin by an electroosmotic pump through the microneedle arrays^22^ or a microtube^33^. However, the fouling of the fluid exchange membrane is a major concern for the delivery of insulin, and the pump can only release insulin with a low-concentration (10–20 U/ml) and a short lifetime^22,33^.

Here, we show for the first time a wearable, rapidly manufacturable, stability-enhancing closed-loop device for diabetes management. The device is built upon polystyrene (PS) hollow microneedles. Polystyrene is cost effective, biocompatible, chemically stable, mechanically strong, and easy-to-manufacture^34^. Polystyrene hollow microneedles can be fabricated via soft lithography. Both the working electrode and the reference/counter electrode are formed by printing a graphene composite ink onto the surface of the microneedles. The entire manufacturing process of sensing electrodes is simple, fast, economical and easy to handle as compared to micro/nanofabrication method. Glucose oxidase (GOD) is then immobilized on the graphene working electrode. To enhance the stability of delivering insulin, an electroosmotic micropump is used and functionalized with an antifouling layer to avoid a performance reduction for delivering insulin. The functionalization is based on polydopamine (PDA), polyethylene glycol (PEG) and bovine serum albumin (BSA). The micropump is further integrated with the microneedles to deliver insulin via the hollow channels of the needles. The microneedle biosensing device detects interstitial glucose, when it is higher than a normal concentration, the micropump is turned on automatically to deliver insulin. Both the sensor and the micropump are operated intelligently by a printed circuit board to lead to a closed-loop function. It is stable, wearable, miniaturized, painless, precise, cost-effective and easy-to-fabricate.

## Results and discussion

### Overall operating mechanism of the system

**Fig. 1:**
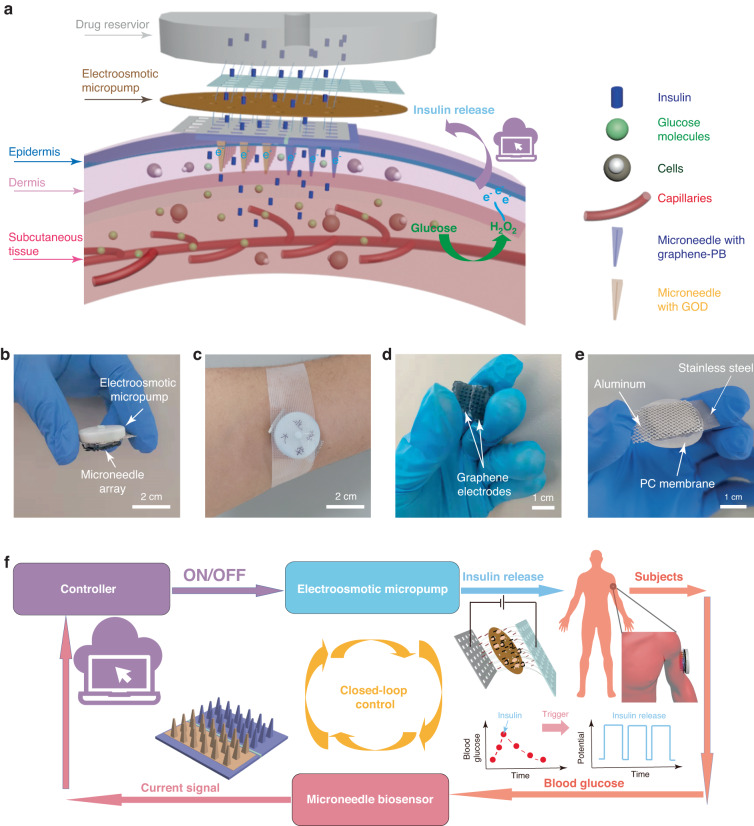
Overall closed-loop patch for diabetes **a** Schematic of the microneedle patch microneedle array on skin. **b** Photograph of the overall system. **c** Photograph of the patch applied on the arm of human subjects. **d** Photograph of a polystyrene (PS) microneedle-based sensor fabricated with two graphene-Prussian blue (PB) electrodes. **e** Photograph of an electroosmotic micropump. **f** Illustration of the overall system’s operating principle

Figure 1a illustrates the overall operating mechanism of an intelligent system placed on the skin for diabetes management. The system includes a polystyrene hollow microneedle array being inserted into the skin dermis layer, a graphene biosensing device coated onto the surface of the microneedles, and a chemically-functionalized electroosmotic micropump. The microneedle biosensing device is painless and causes no bleed for users. Two graphene composite thin-film electrodes are on the surface of the microneedles as the sensing electrode with the immobilization of GOD. The microneedle patch is inserted into the skin dermis layer for detecting interstitial glucose. A chemically-modified electroosmotic micropump with an aluminum mesh as the anode and a stainless-steel mesh as the cathode, is integrated with the microneedle hollow channels. Figure 1b shows a photograph of the entire system, in which both the microneedle sensor and micropump are assembled from a button-shaped 3D printed housing with a diameter of 2.5 cm and a height of 0.5 cm. The shell is a drug reservoir for storing insulin and can hold up to 1.45 ml of insulin solution. The closed-loop system is small enough to be worn by the user (Fig. 1c), which can secure it to the arm or abdomen with a strong medical tape or adhesive bandage to reduce the effects of body movement during indoor and outdoor activities. The microneedle array is only 2 × 2 cm in size and forms an intimate interface with the skin. The two graphene-Prussian blue (PB) electrodes function as working and reference/counter electrodes of the sensor, and they are fabricated by ink coating on the microneedle surface (Fig. 1d). Conventionally, the sensing electrodes are in Au and Ag/AgCl electrodes that are fabricated via physical vapor deposition (PVD)^15^. In comparison, the fabrication process with graphene-PB is cost-effective, simple, and easy-to-perform. For the electroosmotic micropump, the polycarbonate (PC) membrane with nanopores acts as the main component of the pump, allowing insulin to pass through (Fig. 1e). The PDA/PEG/BSA antifouling coatings are applied to the membrane surface subsequently to avoid the contamination of insulin. For the flow of the intelligent system, the microneedle sensing electrode in graphene-PB detects the interstitial glucose level, and the sensing signal is transmitted to a PCB (Fig. 1f). If the measured glucose level exceeds the threshold, the PCB automatically switches on the electroosmotic micropump, and the insulin solution stored in the drug reservoir is released for 10 min, rapidly reaching the interstitial fluid through the hollow channels of the microneedles. The sensor then restarts to detect interstitial glucose. The device performs alternative glucose sensing and insulin delivery until the detected glucose concentration reaches the threshold (Fig. 1f).

The design and functionality of the PCB is the same as we previously reported^22,33^. The PCB is 4.7 × 4.7 cm in size and is connected to the device via wires and to a computer via a USB cable. The computer powers the board, biosensor, and micropump. The user can set the thresholds and operating potentials of the biosensor and micropump and check the current detected by the biosensor on the software interface. For further practical applications in the human body, professional circuit engineers can redesign printed circuit boards to be smaller and consume less power, and transmit data wirelessly to a computer or cell phone, powered by a miniature battery.

In actual human applications, only the microneedle biosensor is in contact with the dermis of human skin. Therefore, the biosensor can be completely sterilized after the electrodes are fabricated, then immobilized with sterile enzymes and stored in a separate sterile environment. Prior to use, the sensor, micropump and PCB electronics can be easily assembled together using materials such as waterproof glue. In addition, due to the low manufacturing cost of microneedle biosensors and micropumps, they can be replaced depending on the functional life of the device, and electronic components such as PCB can be reused.

### Design and fabrication of the microneedle biosensor

**Fig. 2:**
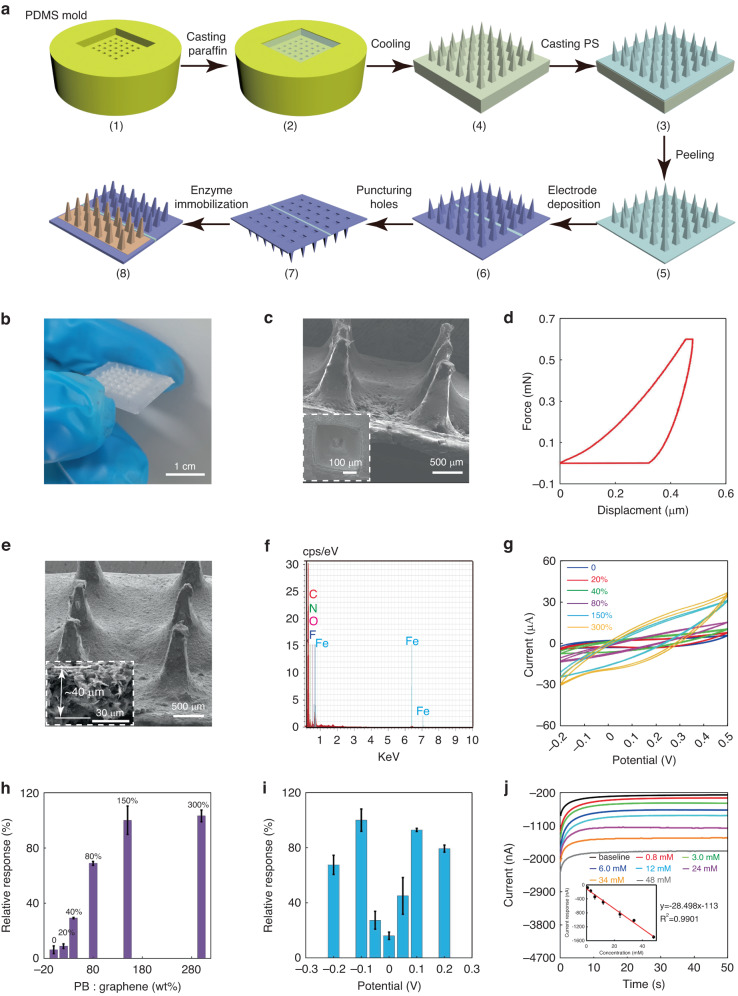
Fabrication of the PS microneedle-based biosensor **a** Schematic of the fabrication process of the sensor. **b** Photograph of the PS microneedle array. **c** SEM images of the side and bottom views of the PS microneedle array. **d** Load-displacement curves for in situ nanomechanical tests on PS substrates. **e** SEM images of the PS microneedles with the deposited sensing electrodes. **f** EDS analysis of the deposited electrode. **g** CV curves of the sensor with the sensing electrodes in different PB: graphene ratios (wt%). **h** Sensitivity of the sensor for detecting H_2_O_2_ with the sensing electrodes in different PB: graphene ratios (wt%) (*n* = 3). **i** Sensitivity of the sensor at different potentials. **j** Current baseline curves and calibration curve (*n* = 3) for measuring H_2_O_2_

Figure 2a exhibits the fabrication of a polystyrene microneedle biosensor. The construction of the microneedle array was based on soft lithography that was economical, non-toxic, and simple to produce. A negative PDMS microneedle mold was obtained by laser engraving (1). The paraffin was melted in the PDMS mold (2), followed by cooling and peeling off to form the paraffin microneedle array (3). A 20% solution of polystyrene was then dropped on the paraffin surface to cover all microneedles and dried over the following 24–48 hours. After that, the polystyrene microneedle array was peeled from the paraffin mold (4–5). Further, the graphene-PB electrodes were deposited on the polystyrene microneedle array (6). Each electrode occupied three rows of microneedles with a length of 2 cm and a width of 9 mm. The tips of microneedles were punctured by the stainless-steel needles to obtain obvious hollow structures (7). Finally, GOD was immobilized on the sidewall of microneedles in the working electrode area for interstitial glucose detection and the biosensor contained 375 U GOD totally (8). From the photographs and SEM images of the PS microneedle array (Figs. 2b, c and S1), it can be seen that each microneedle was shaped like a pyramid with a base diameter of 0.4 mm and a height of 1.2 mm. The dimensions of the square holes at the tip and bottom of the microneedle were 100 μm and 350 μm, and the thickness of the sidewalls was 33 μm (Fig. S2). Figure 2d shows the load-displacement curve of polystyrene substrate measured by an in-situ nanomechanical test system. The elastic modulus of the PS microneedle substrate could reach 2.79 ± 0.02 GPa (*n* = 3) and the hardness reached up to 0.106 ± 0.011 GPa, indicating the high wear-resistance and mechanical stability of the PS microneedle inserted in the interstitial fluid. The force analysis of the microneedle (Microneedle force analysis in Supplemental Material) and the images before and after microneedle insertion (Fig. S3) demonstrate that the microneedle could be inserted into the skin without breaking.

In the composite graphene-PB ink, graphene acts as the conductive electrode, and PB acts as the electron mediator to lower the sensing potential with polyvinylidene difluoride (PVDF) as the adhesive to maintain the stability of the electrode in the liquid. The ink was dropped and coated on the polystyrene microneedle array. As can be seen from the SEM images (Fig. 2e), the microneedle shape was not changed after coating the graphene-PB electrode, and the graphene-PB electrode’s thickness was determined to be 40 μm only. These indicate that the graphene-PB electrode would not affect the insertion performance of the microneedles. From the EDS image in Fig. 2f, C, Fe, N and F elements clearly peaked in EDS point analysis, indicating that the graphene, PB and PVDF were successfully deposited on microneedles. The EDS mapping analysis of the elements on the microneedles, including C, Fe, N and F, confirmed that the graphene, PB (Fe_4_[Fe(CN)_6_]_3_) and PVDF ([C_2_H_2_F_2_]n) were mixed uniformly and distributed evenly on the microneedles (Fig. S4). In addition, when there was no PB in the electrode, the layer structure of graphene was obvious; when the ratio was 150%, the vacancy of graphene was occupied by PB (Fig. S5). Further, the effect of the graphene to PB ratio (wt%) was studied. From the CV curves in Fig. 2g, with the increase of the ratio from 0 to 150%, the current window was widened significantly. When the ratio increased from 150% to 300%, the current window was changed obviously. Similarly, electrochemical impedance spectroscopy (EIS) analysis was performed for the sensing electrode, and it showed that its electron transfer resistance decreased as the ratio increased from 0 to 150%, indicating that the electron exchange efficiency was enhanced with an increasing PB content and saturated at a ratio of 150% (Fig. S6). Moreover, with different PB: graphene ratios, the sensitivity of the sensor for detecting H_2_O_2_ was studied. It was obvious that the sensor was most sensitive for detecting H_2_O_2_ when the ratio was 150% and 300% (Fig. 2h). These results indicate that a graphene to PB ratio of 150% was an optimized ratio for the ink and can be used for further studies. The working potential was studied, as shown in Fig. 2i. The highest relative response was obtained at a potential of −0.1 V, which was selected for further sensing studies.

The graphene-PB microneedle electrode in PBS showed an excellent stability in PBS, and there is no significant change of the microneedle surface after being immersed in PBS for two weeks (Fig. S7). The graphene-PB electrode was also very stable after multiple CV measurements (Fig. S8). Since the materials for both working and reference electrodes were graphene-PB, the reference electrode cannot provide a stable potential (Fig. S9). The baseline current was recorded upon different concentrations of H_2_O_2_, and the calibration curve was established according to the final current value of each curve (Fig. 2j). The graphene-PB electrode showed an excellent detection of H_2_O_2_. The baseline currents at 50 s increased linearly with the increasing H_2_O_2_ concentration from 0.8 mM to 48 mM with a slope of 28.498 ± 0.605 nA/mM (*n* = 3). The results indicate that the sensor based on two graphene-PB electrodes sensor was able to perform an excellent detection of glucose, since H_2_O_2_ was the end product of the enzymatic reaction by GOD.

### In-vitro sensing of the microneedle graphene-PB biosensor

**Fig. 3:**
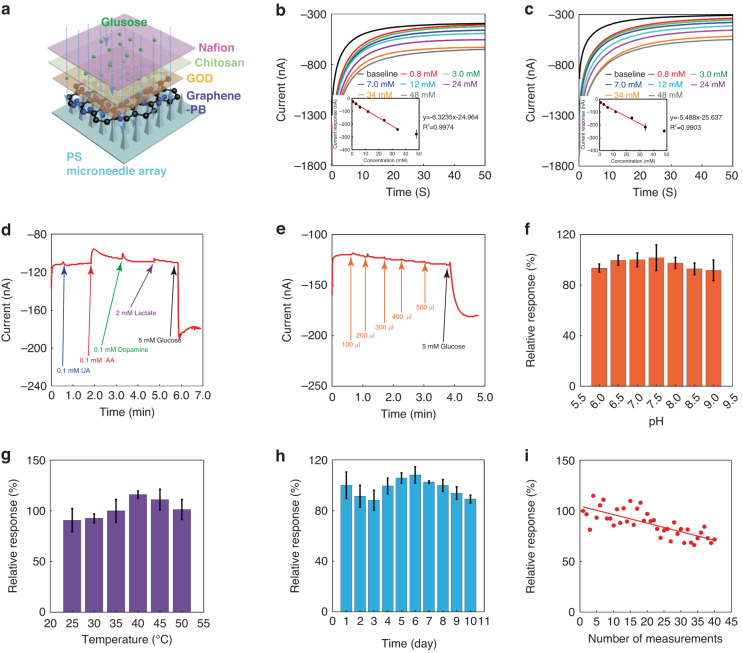
In-vitro performance of the biosensor to detect glucose **a** Layer structure of the microneedle sensing electrode. **b** Current response curves for glucose in PBS. Inset: calibration curve (*n* = 3). **c** Current response curves for glucose in simulated interstitial fluid. Inset: calibration curve (*n* = 3). **d** Selectivity study of the microneedle sensor (UA: uric acid, AA: ascorbic acid). **e** Study of the effect of insulin Aspart amounts to the sensor. **f** Study of the effect of pH on the sensor (*n* = 3). **g** Study of the effect of temperature on the sensor (*n* = 3). **h** Study of the effect of storage time on the sensor (*n* = 3). **i** Study of the effect of repeatability on the sensor

Figure 3a illustrates the multilayer structure on the working electrode. To complete the construction of the microneedle, GOD, chitosan and Nafion were deposited onto the graphene-PB working electrode sequentially, and each layer was smooth with homogeneous morphologies (Fig. S10). After the immobilization of GOD, the current values in the CV curves became smaller, and the electron transfer impedance value became larger (Fig. S11), indicating the successful attachment of the enzyme on the sensing electrode. The chitosan and Nafion membranes were deposited on the graphene-PB electrode respectively for promoting the electrode stability, increasing the in-vivo biocompatibility and resisting the electroactive interferences in interstitial fluid.

The CV curves of the graphene-PB microneedle sensing device showed similar shapes at different scanning rates, and the current peaks were raised with an increasing scanning rate (Fig. S12). A linear sensing range of 0.8-34 mM with a sensitivity of 6.3235 ± 0.3410 nA/mM can be observed for detecting glucose in PBS (Fig. 3b). Similarly, the calibration curve in Fig. 3c demonstrated a linear detection range of 0.8-34 mM for glucose in simulated interstitial fluid with a sensitivity of 5.4880 ± 0.8210 nA/mM (*n* = 3). The exclusion of interferences is critical to achieve a high accuracy for detecting glucose in-vivo. The effect of the electroactive interferences on sensing glucose was studied, and the current response to 5 mM glucose was significantly higher than that induced by uric acid (0.1 mM), ascorbic acid (0.1 mM), dopamine (0.1 mM) and lactate (2 mM) (Fig. 3d). Therefore, the interfering effect of these electroactive substances was neglectable. In addition, insulin was delivered via the hollow microneedles into the dermis layer, which may not be adsorbed efficiently enough and stay around the microneedle. This may affect the accuracy for sensing interstitial fluid. The current response to 5 mM glucose was much larger than the signals caused by various insulin dosages (100 U/ml) (Fig. 3e). These findings indicate that the accumulation of insulin solution around the microneedles had a neglectable effect on sensing interstitial fluid.

Further, the environment, human activities and diseases could sharply change the pH and temperature of interstitial fluid^35–38^. Thereby, it was essential for the sensor to be able to resist these changes and keep stable under different circumstances. From Fig. 3f and g, the sensor showed the excellent pH and temperature stabilities, with the smallest relative responses of 91.73% at pH 9 and 90.86% at 25 °C. The biosensor showed an excellent storage stability and could keep 89.11% of its initial response for over 10th day (Fig. 3h), demonstrating that the measure effectiveness of the biosensor could be maintained for more than 10 days. The measurement repeatability of the biosensor was also studied as well, and it could maintain 71.39% of its original response after the consecutive 40 times glucose detection (Fig. 3i). The variation of relative response in different environments and continuous measurements may be due to the fluctuation of enzyme activity and the depletion of graphene-PB in multiple consecutive measurements. To further improve the accuracy of the biosensor, temperature and pH sensors could be integrated into the biosensor in future applications, and the glucose detection results of the biosensor could be calibrated based on changes in temperature and pH. The glucose detection results can also be calibrated based on changes in the relative response of the biosensor when performing multiple consecutive measurements. In summary, all these results indicate that the biosensor has relatively excellent accuracy and stability to perform in-vitro glucose detection under various conditions.

The variation of relative response in different environments and continuous measurements may be due to the fluctuation of enzyme activity and the depletion of graphene-PB in multiple consecutive measurements. To further improve the accuracy of the biosensor, temperature and pH sensors could be integrated into the biosensor in future applications, and the glucose detection results of the biosensor could be calibrated based on changes in temperature and pH. The glucose detection results can also be calibrated based on changes in the relative response of the biosensor when performing multiple consecutive measurements.

### Design and fabrication of the electroosmotic micropump

**Fig. 4:**
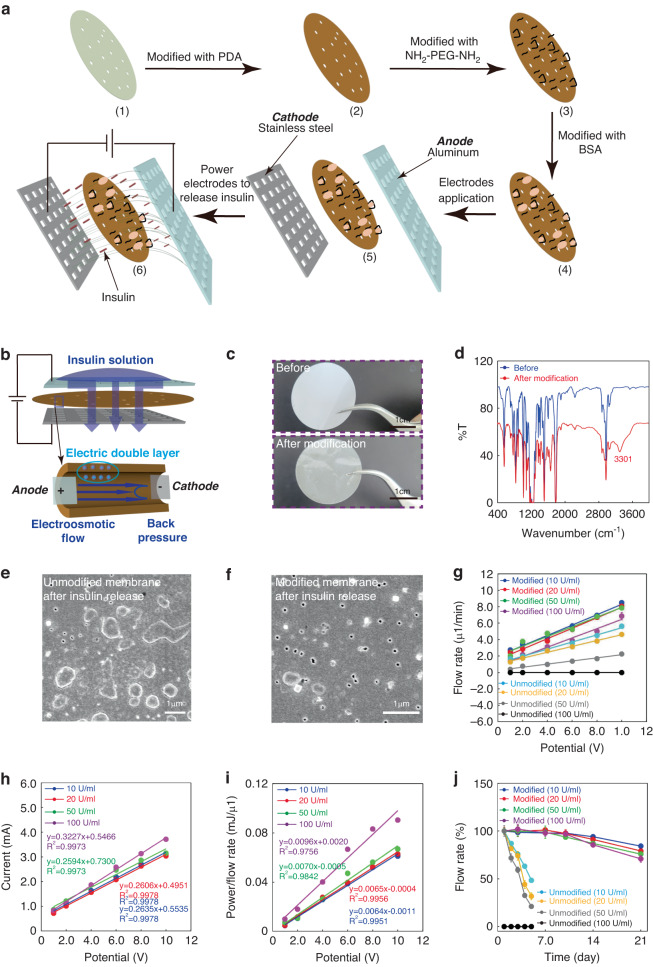
Fabrication of the electroosmotic micropump **a** Schematic of the electroosmotic micropump. **b** Operation mechanism of this electroosmotic micropump. **c** Photograph of the PC membrane before and after modification. **d** Infrared-spectrum of the PC membrane before and after modification. **e** SEM image of the unmodified PC membrane after insulin release. **f** SEM image of the modified PC membrane following the release of insulin. **g** Flow rates for delivering insulin of the micropump at different potentials (*n* = 3). **h** Currents of the micropump at different potentials for delivering insulin. **i** Power/flow rate of micropump for delivering insulin at different potentials. **j** Stability of micropump for releasing different insulin solutions with different storage time (*n* = 3)

A miniaturized and stable micropump is highly desired for the delivery of insulin. To achieve this goal, an electroosmotic micropump with an antifouling coating was studied. PEG is the hydrophilic polymer that could form the osmotic barrier to inhibit the adsorption of proteins^39,40^. To conjugate PEG to the PC membrane, a PDA was used as an adhesive layer. BSA was further used to remove the protein adsorption. Figure 4a illustrates the design and fabrication of the electroosmotic micropump. The PC membrane (1) was nano-porous, and further modified with PDA (2), NH_2_-PEG-NH_2_ (3) and BSA (4) layers. The PDA/PEG/BSA coating can decrease the fouling significantly to further enhance the stability and lifetime. Then, the anode and cathode, aluminum mesh and stainless steel mesh, respectively, are assembled with the modified PC membrane to build the overall pump (5). Aluminum was inert relatively and could resist corrosion during the injection of insulin, to further increase the lifetime of the micropump. The stainless-steel mesh had a great flexibility and can contact the microneedle array closely. A constant DC voltage was applied between the aluminum mesh and the stainless-steel mesh to power the micropump (6). The working mechanism of the micropump is illustrated in Fig. 4b. The nanopores in the PC membrane were the transport channels for the insulin solution^41,42^. An electric field was formed between the two electrodes across the membrane. On the interface between solid and liquid, a double electron layer (DEL) was formed by the surface charge. The charges in the diffusion layer of DEL would migrate in the same direction as the electric field, which further drove the directional migration of surrounding liquid to form the electroosmotic flow^42,43^. The thickness of the PC membrane is about 7-22 μm, and when a constant voltage of 10 V is applied to the membrane, the generated electric field across the membrane is ~10^6 ^V/m. It has been reported that the insulin molecule is able to withstand electric fields as high as 10^8^ V/m^44^. Therefore, electroosmotic micropumping does not disrupt the structure of the insulin molecule, nor does it affect the behavior of the insulin molecule. Figure 4c exhibits the camera image of a PC membrane before and after being modified with PDA/PEG/BSA. The membrane after modification displayed a clear brown color, which was a feature color of the PDA. As can be seen from the SEM image of the PC membrane in Fig. S13a, multiple nanopores with diameters of 200 nm were scattered on the membrane’s surface. After modifications, the roughness of the membrane surface was improved (Fig. S13b). Figure 4d illustrates the infrared spectrum of the PC membrane before and after modification. There was an obvious peak at 3301 cm^-1^ after the modification, demonstrating the -NH_2_ and -OH groups from the NH_2_-PEG-NH_2_ and PDA. This demonstrated that the membrane was successfully modified with PDA and PEG.

The transport of deionized water and varied concentrations of insulin over unmodified or modified membranes was studied (Fig. S14). By using the unmodified membrane to release insulin with a concentration of 100 U/ml, it was observed that there was almost no insulin released to the bottom stainless-steel electrode within 40 min. By using the modified membrane, when there was no voltage applied to the membrane, no liquid transport was observed, indicating that the liquid flow was not affected by gravity. With an applied voltage of 10 V across the membrane, deionized water and insulin with a concentration of 100 U/ml were gradually transported across the membrane. Figure 4e, f display the SEM images of the unmodified membrane and modified membrane after pumping insulin (100 U/ml). It can be seen that there were many insulin crystals adhered on the unmodified membrane after pumping insulin (Fig. 4e), and no obvious insulin crystals on the modified membrane (Fig. 4f).

By applying the same voltage across the membrane, the chemically modified micropump showed a much higher flow rate than the non-modified pump when releasing insulin at concentrations of 10 to 100 U/ml (Fig. 4g). In addition, for a same concentration and a same membrane, the insulin release rate increased with increasing applied voltage. As the insulin concentration increased from 10 to 100 U/ml, the flow rate decreased from approximately 0.63 to 0.57 μl/(min·V) for the modified membrane, and from 0.43 μl/(min·V) to 0 for the non-modified membrane. The results demonstrate that the modified membrane can release insulin more efficiently, especially for insulin with a high concentration.

The electrical conditions for the modified micropump were further studied, as shown in Fig. 4h, i. The generated current across the membrane increased with the applied voltage linearly. When the insulin concentration changed from 10 to 100 U/ml, the slope varied slightly within a small range from 0.26 to 0.32 mA/V, as can be seen from Fig. 4h. The power consumptions were studied for the membrane with different concentrations of insulin. The differences of the power consumptions were in the low millivolt range and varied slightly between 7.57% to 16.17% for the insulin concentrations from 10 to 100 U/ml (Fig. S15). The power consumption per flow rate increased with increasing potential (Fig. 4i), indicating that a higher potential can enhance the efficiency of the micropump. Since the ratios of the flow rate for 100 U/ml insulin to the flow rate for 10 U/ml insulin ranged from 1.24 at 10 V to 1.97 at 1 V (Fig. 4g), the ratio of the power consumption per flow rate for 100 U/ml to that for 10 U/ml was in the range from 1.5 to 2.1. The results demonstrated that to deliver a same amount of insulin, a concentration of 100 U/ml required a higher power consumption.

The stability of insulin delivery over a 3-week period was studied with and without surface modification of the micropump. Figure 4j demonstrates that the surface modification can enhance the working stability significantly. The non-modified membrane cannot deliver insulin with a concentration of 100 U/ml. Although the non-modified membrane can deliver the insulin solutions with the concentrations from 10 U/ml to 50 U/ml, the flow rate dramatically decreased. For example, on the fifth day, the flow rate decreased to 48.44% of the initial flow rate (at the first day) for 10 U/ml, 31.78% for 20 U/ml, and 21.01% for 50 U/ml. In comparison, the flow rate for the modified membrane can maintain about 97% of the initial flow rate on the 10^th^ day for all concentrations of insulin. Even on the 21st day, the flow rate can be 84.17% of the initial flow rate for 10 U/ml, 79.08% for 20 U/ml, 75.77% for 50 U/ml, and 70.83% for 100 U/ml. These results demonstrate that the surface modification on the PC micropump enabled the membrane to possess an excellent working stability and significantly enhance its pumping performance over a longer time.

### In-vivo sensing in diabetic rats

**Fig. 5:**
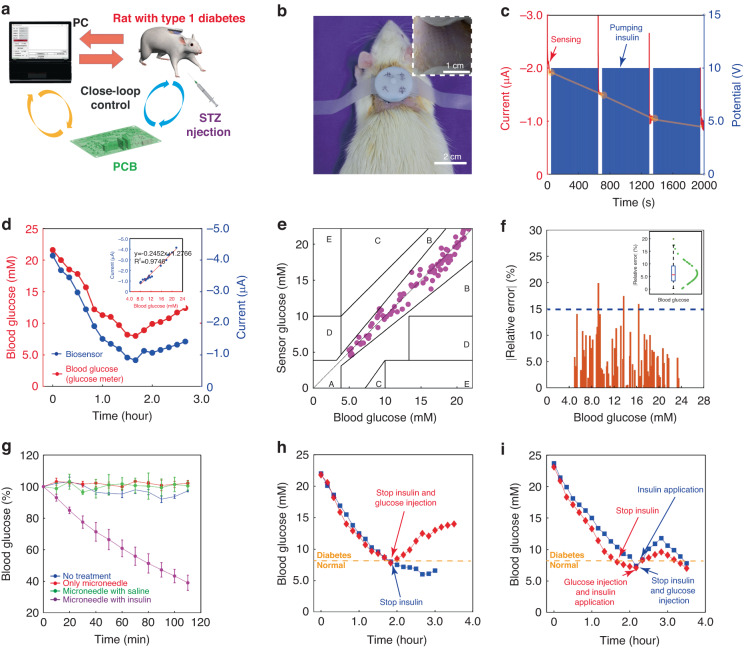
In-vivo sensing in diabetic rats **a** Schematic of the microneedle patch applied to the skin of a diabetic rat. **b** Photograph of the patch applied to the rat. Inset: pressure marks on the back skin of a rat after applying the patch for 5 min. **c** Operating model of the patch applied to the rat. **d** Blood glucose change over time measured by a commercial glucometer and the microneedle sensor. **e** Clarke rigor grid of blood glucose levels measured by a commercial glucometer and the microneedle sensor (the results were collected from six completely different diabetic rats). **f** Sensing errors of the microneedle sensor by comparing to the commercial glucometer. **g** Blood glucose change (%) with and without microneedle and insulin. **h** Blood glucose levels versus time after the suspend of the system with and without a glucose injection. **i** blood glucose levels versus time with a continuous closed-loop management under different conditions

To further evaluate the function and performance for diabetes management, the microneedle device was applied to diabetic SD rats. Before experiments, the diabetic SD rats model was established by intraperitoneal injection of STZ in step with the protocol^45^. The device was applied to the skin of a diabetic rat, and the microneedle sensor and the electroosmotic pump were powered and controlled by a PCB (Fig. 5a). The rat’s back was selected to fix the minisystem (Fig. 5b). After withdrawing the minisystem from the rat’s skin, the pressure mark left on the skin showed the pore patterns to be highly consistent with the microneedle arrays. From the hematoxylin and eosin-stained penetrated rat’s back skin section after removing the microneedle, a pyramid pore was formed on the skin (Fig. S16). These demonstrated that the rat skin can be penetrated by the microneedles successfully. The weight of the graphene-PB electrode for each biosensor was measured to be 4.50 ± 0.49 mg (*n* = 3), and the loss of electrode weight after 3 hours of insertion into the skin was 0.09 ± 0.03 mg. Considering the error in weight measurement using a balance (accuracy: 0.01 mg), the loss of graphene electrode coating after skin piercing was almost negligible. To evaluate the in vivo biocompatibility of the biosensor, a rat skin irritation test was performed (Fig. S17). After 6 days of using the biosensor, no severe skin reactions such as erythema and edema were observed, which was mainly due to the excellent biocompatibility of the chitosan and Nafion membranes deposited on the biosensor.

An alternative two-step control mode was adopted for achieving an automatic closed-loop diabetes management control (Fig. 5c). A cycle was totally 650 s, including 50 s’ sensing and 10 min’s insulin delivery. The microneedle biosensing device was operated at -0.1 V for 50 s for measuring interstitial glucose at the beginning (the first red line), and the current at the 50 s was processed by a PCB to determine if blood glucose level was above the threshold (8.3 mM)^45^. If the result exceeded this value, the micropump was subsequently powered at 10 V to release insulin (100 U/ml) stored in the top reservoir (first blue line) for 10 min, and the insulin solution would quickly reach the interstitial fluid through the hollow channels of the microneedles. As shown in Fig. 4g, the in-vitro release rate of insulin from the micropump was 6.85 μl/min. After that, the sensing device was operated again to measure glucose (the second red line). The PCB operated an alternative process of repeated sensing and pumping until the blood glucose level fell below a critical value, at which point insulin delivery was stopped, and only the sensing of interstitial glucose was performed only once every 10 min. The separate glucose sensing and insulin pumping steps could decrease the interfering effect from insulin in glucose sensing.

To assess the accuracy of the microneedle sensor, we established a correlation between the currents of the microneedle sensor and the blood glucose levels from clinically approved commercial glucose meters. As seen in Fig. 5d, the trend of the current matches well with the fluctuation of blood glucose, and a clear linear correlation is observed with a slope of 0.2472 μA/mM and an R^2^ of 0.9746. The Clarke error grid illustrates the difference between blood glucose levels determined by the sensor and the commercial glucose meter (data from 6 rats) (Fig. 5e). There are 89 points in both clinical safety zones A and B, which indicates that the microneedle sensor is accurate. Compared to the glucometer values, the microneedle sensor values ranged from 0.179% to 19.896%, with a MARD (mean absolute relative difference) of 6.208% ± 4.259% and 75% of the points below 9.16% (Fig. 5f). These results are in accordance with the ISO 15197:2013 standard for glucose sensing accuracy^46^, indicating that the sensor is capable of responding correctly and reliably to changes in blood glucose. Compared to commercially available blood glucose meters, the biosensor has the potential to enable accurate continuous glucose monitoring, avoiding the burden and inconvenience caused by frequent finger-pricking blood collection.

Figure 5g shows the study of the hypoglycemic effect in four groups of diabetic rats. The four conditions were (1) no treatment (blue line), (2) microneedle application only (red line), (3) saline delivery with the microneedle device (green line), and (4) insulin delivery with the microneedle device (purple line) (each from three rats). For the first three controls, there was almost no reduction in blood glucose levels, as shown by the blue, red and green lines in Fig. 5g. The results indicate that the device itself and saline had no hypoglycemic effect on blood glucose. In contrast, when the device delivered insulin through the internal channel of the microneedle, blood glucose levels dropped significantly to 39.15% of its starting amount within 110 minutes. These findings demonstrate that insulin can be effectively delivered into the skin through the microneedle system, providing excellent hypoglycemic control in diabetic rats.

Figure 5h demonstrates the blood glucose changes when the intelligent system lowers the blood glucose of diabetic rats to the normal range. In the first case (blue line), the blood glucose level of the diabetic rats was reduced from 22.0 mM to 8.0 mM (<8.3 mM) in approximately 110 minutes due to the operation of the intelligent system for monitoring blood glucose and pumping insulin (Fig. S18). Then, insulin infusion was manually stopped. Due to the previous insulin infusion, the blood glucose level was able to remain within the normal range (6.6 mM at the 180th minute). In the second case, similar results were obtained using the closed-loop device (red line), with a decrease in blood glucose from 21.8 mM to 7.8 mM at 110 min. Then, insulin release was stopped manually while glucose solution (1 g/kg) was injected intraperitoneally to simulate food intake and raise blood glucose levels. Thereafter, due to the large amount of glucose injected at once and the absence of continuous insulin administration, blood glucose levels gradually increased, reaching 14.0 mM at the 210th min. These results suggest that the intelligent system was able to bring blood glucose levels down to the normal range, while glucose intake would have raised blood glucose levels to diabetic levels.

Figure 5i exhibits the effect of a continuous operation of the intelligent system on blood glucose of diabetic rats with a glucose intake. The first scenario (blue line) studied the effect of a glucose intake with setting the start of insulin injection at a glucose concentration of above 8.3 mM. When the blood glucose level rose beyond the threshold (8.3 mM), the system was stimulated to start the injection of insulin automatically. As can be seen from the Figure, a diabetic rat’s blood glucose level dropped from 23.7 mM to 7.4 mM in 130 min with the use of the intelligent system only. After that, glucose was administered intraperitoneally, which further raised the blood glucose level. At 140 min, when the blood glucose level rose to 8.4 mM (greater than 8.3 mM), the intelligent system started administering insulin. During the initial phase of insulin infusion, the blood glucose was still rising because of the tiny amount of insulin delivery. Gradually, with the increased quantity of insulin being administered in vivo, the blood glucose gradually stopped rising and peaked at 11.8 mM at the end of 170 min, after which it began to fall until it was below the critical value (8.3 mM) at 210 min.

For the second scenario (red line), the different timing of glucose and insulin injections to diabetic rats was studied. The operation of the intelligent system caused the blood glucose level of the rat to drop from 23.1 mM to 7.6 mM after 100 min, at which point insulin delivery was stopped. At 130 min, the blood glucose level dropped to 7.1 mM, when glucose was administered intraperitoneally. At the same time, the critical value for blood glucose was adjusted to be its current glucose level (7.1 mM) and insulin was started to be injected automatically at 7.1 mM glucose. As shown in the red curve, the blood glucose level peaked at 9.6 mM at 170 min and then rapidly fell to 7.0 mM at 210 min.

From the above results and the comparison of the different curves in Fig. 5h, i, it appears that after using the closed-loop patch, although the blood glucose level rose above the normal range (blue and red curves in Fig. 5i), the peak of blood glucose was not as high as it would have been without operating the closed-loop device (red curve in Fig. 5i). When insulin was started at blood glucose levels below the threshold (red curve in Fig. 5i), the peak after the blood glucose rise was not as high as when the device was started at the threshold (blue curve in Fig. 5i). This suggests that early insulin intake accelerates the breakdown of injected glucose by the blood, thus further lowering blood glucose. These results suggest that a closed-loop device could provide better control for diabetes management, and that adjusting the threshold to a lower value could reduce the rise in blood glucose and prevent it from reaching a high level if the user were to eat a meal. Compared with this intelligent system, the traditional manual insulin injection has obvious disadvantages. If too much insulin is injected manually at once, it can cause significant hypoglycemia (blue line of Fig. S19); if too little insulin is injected, blood glucose rises sharply after glucose intake (red line of Fig. S19).

Due to the limitations of the anesthesia machine, rats are more likely to die if the anesthesia lasts longer than 4-5 hours. As a result, the insulin release cycle and duration time are relatively short in the in-vivo experiments. To elevaluate the effectiveness of the system for long-term diabetes management in rats, this system was applied on the same diabetic rat for three consecutive days for about five hours each day, and the rat showed similar trends in daily blood glucose (Fig. S20). After using the system, the blood glucose of the rat would quickly drop to the normal level in about 2 hours at the beginning, and after intraperitoneal injection of glucose, its blood glucose would also quickly return to the normal range. The system was also effective in managing late stage rats that were modeled for more than one month (Fig. S21). The reduced insulin sensitivity in these rats may account for the slower rate of decline in blood glucose levels and the longer administration period in these rats. All these findings suggest that the system is capable of successfully managing blood glucose levels in diabetic rats on an autonomous closed-loop basis. In practical human applications, more complex algorithms than the existing ones (which release insulin when blood glucose reaches a set value) are needed to avoid fluctuations in blood glucose levels outside of the safe range due to imprecise timing and amount of insulin injections.In further practical applications, professional algorithm engineers are needed to further improve the control algorithm to make it more intelligent and smarter, such as by introducing the proportional-integral-derivative (PID) controller and the model predictive control (MPC) approach^47,48^.

## Conclusion

We have demonstrated a wearable, rapidly manufacturable, stability-enhancing closed-loop patch for diabetes management. A graphene-PB ink was prepared and deposited on the microneedles to function as both working and reference/counter electrodes, enabling the process fast, inexpensive, and suitable for mass production. The electroosmotic pump modified with a PDA/PEG/BSA antifouling layer can significantly improve its stability and maintain the flow of released insulin (100 U/ml) at more than 70% of the initial flow for more than three weeks. This system is advantageous over other existing commercial or reported closed-loop systems for being wearable, small, lightweight, low-cost, and manufactured quickly. Although these results are promising, the control algorithm should be improved and the performance of this system should be evaluated in human diabetes patients before further practical applications. A more compact PCB or chip is needed to make the system more wearable, and data should be transmitted wirelessly to mobile devices, such as smartphones, eliminating the need for a computer connection. In addition, the processing of the whole device can be further simplified to improve its manufacturing feasibility and reduce the cost, e.g., the hollow microneedle arrays can be directly printed using 3D printing techniques, the graphene-Prussian blue electrodes and the enzyme immobilization can be printed on the sidewalls of the microneedles using aerosol jet printing techniques. The integration of the biosensor and the micropump components can be accomplished directly using industrial robots. This work may expand the fundamental studies on novel devices for diabetes management, as well as advance their practical applications in diabetes patients.

## Materials and Methods

### Fabrication of PS microneedle array

The solid polystyrene (PS) was dissolved into dimethylformamide (DMF) completely in an oven at 90 °C to form a 20% (wt%) PS solution. Then, the solid paraffin covered on the concave PDMS mold and melted at 90 °C, following by being cooled down at room temperature. The PS solution of ~500 μl was then applied to the paraffin mold to cover all microneedles and dried on a hot plate at 45 °C for 24-48 h. The paraffin mold was eventually removed from the PS microneedle array. The array had 6 × 6 microneedle arrays, each with a pyramidal shape, a base width of 400 µm and a height of 1.2 mm, and a spacing of 2 mm between microneedles. After fabricating the electrodes, the tips of microneedles were punctured by the stainless-steel needles to obtain the hollow structures.

### Fabrication of the biosensor

The PS microneedle array biosensor was composed of two graphene-Prussian blue (PB) electrodes and each electrode occupied 3 microneedle columns. The graphene-PB electrode was prepared by combining 40 mg graphene powders, 60 mg PB powders and 40 mg polyvinylidene fluoride (PVDF) powders in a mortar to achieve a fine enough grind. Then, the mixed powders were dissolved in a 3 ml of ethanol, and homogenized by an ultrasonic device for 15 min. Then, a 500 μl graphene-PB-PVDF/ethanol solution was dropped on the surface of the PS microneedle array to form two electrodes. Prior to sensing measurements, the microneedle sensor was immersed in 300 μl of 0.1 M KCl/HCl, and a CV scan from -0.2 V to 0.5 V was performed at a scan rate of 50 mV/s to stabilize the graphene-PB electrode.

The preparation of the microneedle biosensing electrodes with various PB to graphene ratios (wt%) was performed to maximize the sensing performance. The sensor was submerged in a 0.1 M KCl/HCl solution to perform the CV scanning and the electrochemical impedance spectroscopy (EIS) measurements from 1 × 10^-2^ to 1 × 10^5^ Hz. The amperometric sensing of H_2_O_2_ was conducted for each biosensor at a working potential of −0.1 V in comparison to the reference electrode, and their sensitivities were compared.

Glucose oxidase (GOD) was required to be immobilized on one graphene-PB electrode for the glucose detection. The hydrophilic electrode surface was obtained by treating the biosensor in the UV ozone for 10 min before the enzyme immobilization. A mixture containing of GOD (7.5 μl, 50 U/μl) solution, bovine serum albumin (BSA) solution (7.5 μl, 3%) and a diluted glutaraldehyde (15 μl, 2%) was dropped onto one graphene-PB electrode surface. The sensing device was kept in a 4 °C refrigerator overnight. After this, two electrodes were coated with a 30 μl of 1% chitosan solution (in 2% acetic acid) and dried at 4 °C for 2–4 hours. Finally, a 30 μl of a Nafion (1%) solution was coated onto the two electrodes and dried in the refrigerator at 4 °C for 2–4 hours before the sensing measurements.

### Characterization of sensing performance

In the sensing measurements, a constant potential at −0.1 V was supplied between the working and reference electrodes by a potentiostat at room temperature. The working electrode was one graphene-PB electrode immobilized with GOD, while the counter/reference electrode was the other graphene-PB electrode. Glucose assays were performed in phosphate buffer (PBS) (50 mM pH 7.0), and simulated interstitial fluid. Prior to the experiment, the biosensor was initially submerged for 30–60 min at room temperature into a 300 μl of PBS to activate GOD. During the sensing process in PBS, the biosensor was submerged into a 300 μl of PBS containing various concentrations of glucose. The current-versus-time curves for each concentration were recorded after 50 s’ measurement. The calibration curve was plotted between the final current points and the glucose concentrations.

In the preparation of the simulated interstitial fluid, alginic acid sodium was dissolved into 0.1 M KCl at 45 °C for 4 h to obtain a 1.5% w/v viscous solution, followed by dissolving a certain amount of glucose powder. After that, the solution was fully covered by 0.2 M calcium chloride (CaCl_2_) and stored at 4 °C overnight to obtain a hydrogel containing glucose. The hydrogel layer was penetrated by the microneedle array and the glucose concentration was also measured for 50 s.

The sensor was submerged into a 300 μl of PBS to carry out the amperometric measurement in order to evaluate the impact of various electroactive interferences and varying quantities of insulin solutions (100 U/ml) on the sensing performance. When the current was relatively stable, 12 μl of uric acid, ascorbic acid, dopamine and lactate solutions, different volumes of insulin solutions, and a 12 μl glucose solution were dropped into PBS. The generated current responses were compared.

To investigate the pH effect on sensor activity, the sensors were immersed in PBS at pH 6.0, 6.5, 7.0, 7.5, 8.0, 8.5 and 9.0 and the sensing responses to glucose (4 mM) were recorded. To investigate the temperature effect on sensor activity, the sensing responses to glucose (4 mM) were recorded at different temperatures using a hot plate. To study the effect of storage on the stability of the sensor, the sensing responses to glucose (4 mM) were tested three times a day for more than ten days. When not in use, the sensor was stored in PBS at 4 °C. The sensing responses to glucose (4 mM) were measured 40 times continuously to assess the repeatability of the sensor.

### Preparation and characterization of electroosmotic micropump

A polycarbonate (PC) membrane modified with polydopamine (PDA), polyethylene glycol (PEG), and BSA coatings is the key element for the electroosmotic micropump with an anode mesh and a cathode mesh on both sides. The anode was made of aluminum mesh, and its cathode was made of 304 stainless steel mesh. These meshes had a 1.5 cm width and a 2 cm length, and the PC membrane had a 2.5 cm diameter.

For modifying the PC membrane, the membrane was first immersed into the 2 mg/ml dopamine hydrochloride solution (dissolved in 1x PBS at pH 8.5) for 3 h at room temperature. After that, the deionized water was used to completely rinse the newly modified membrane. Then, the PDA modified membrane was stored in 20 mg/ml NH_2_-PEG-NH_2_ (MW = 20,000) solution (dissolved in 1x PBS at pH 8.5) for 24 h at 37 °C. The PDA/PEG modified membrane was then incubated in 10 mg/ml BSA (dissolved in PBS at pH 7.4) at room temperature for 24 hours. During the measurement of the flow rate, the insulin released from the micropump was collected every 5 minutes and its weight was determined using a balance. The flow rate was then calculated by converting the weight of the insulin solution to a volume based on its density and dividing by the release time. At a specific potential, each flow rate was measured 3 times.

### Rats’ experiment

The Research Ethics Committee of Peking University First Hospital approved the experiments with diabetic rats (Approval Number: 2021112). To carry out the rat’s experiments, male Sprague Dawley (SD) rats with weights of 150–200 g were acquired from Sibeifu Beijing biotechnology company (License Number: SCXK(Jing) 2019-0010). Streptozotocin (STZ), a drug that could selectively kill pancreatic islet β-cells, was intraperitoneally injected into all of the healthy rats to induce diabetes. Rats were fasted for 6 to 8 h, and then STZ in citrate buffer (50 mM, pH 4.5) was administered intraperitoneally to the rats at a dose of 65 mg/kg.

Throughout the experimental period, all rats were housed in individually ventilated cages under pathogen-free conditions. On the tenth day the rats were given a 6–8 hour fast and blood samples were taken from the tail vein for glucose measurement to determine if the rats had reached diabetic status.

The diabetic rats were fasted for about 8 h and then anesthetized with an isoflurane anesthesia machine prior to the study of the closed-loop device. The insertion site of the skin on the back of the rats was scraped and cleaned before the device was mounted on the rats. The device was then mounted on the skin of the rat and allowed to warm up for 30 min. After that, the sensor detected the blood glucose level for 50 s, and if the sensed result exceeded 8.3 mM, the micropump was activated and insulin was injected at a rate of 100 U/ml for 10 min. After this, the sensor performed another blood glucose test. Until the blood glucose level reaches a healthy value, sensing and pumping were performed alternately. Thereafter, only blood glucose was measured, at which point insulin was discontinued. During sensing, the blood glucose level of the tail vein sample was measured by a clinically approved commercial glucose meter.

### Supplementary information

Supplemental Material
