# Supplementary material for: A wearable, rapidly manufacturable, stability-enhancing microneedle patch for closed-loop diabetes management
Source: Microsyst Nanoeng. 2024 Aug 19;10:112. doi: 10.1038/s41378-024-00663-y (PMC11333613; doi:10.1038/s41378-024-00663-y)
Supplement: Supplementary file 1 — Supplemental Material [file 41378_2024_663_MOESM1_ESM.docx]

Supplemental Material for

A Wearable, Rapidly Manufacturable, Stability-Enhancing Microneedle Patch for Closed-Loop Diabetes Management

Yiqun Liu^1^, Li Yang^2*^, Yue Cui^1*^

^1^ School of Materials Science and Engineering, Peking University; First Hospital Interdisciplinary Research Center, Peking University, Beijing 100871, P.R. China

^2^Renal Division, Peking University First Hospital; Peking University Institute of Nephrology; Key Laboratory of Renal Disease, Ministry of Health of China; Key Laboratory of Chronic Kidney Disease Prevention and Treatment (Peking University), Ministry of Education, Beijing 100034, P.R. China

E-mail: [ycui@pku.edu.cn](mailto:ycui@pku.edu.cn), [li.yang@bjmu.edu.cn](mailto:li.yang@bjmu.edu.cn)

**Microneedle force analysis**

When the microneedle is inserted into the skin, it was under five ultimate loads during: the compression force, the buckling force, the free bending force, the constrained bending force and the maximum shear force^1^. The most important of these is the maximum buckling force because it has the smallest value:

The maximum buckling force that the microneedle could be calculated as according to the mathematical model introduced by Smith^2,3^:

$$F_{cr}=\frac{E\pi^{2}}{2L^{3}}\int_{0}^{L} I\left( z \right).{cos}^{2}\left( \frac{\pi z}{2L} \right)dz$$

where $F_{cr}$ is the critical buckling load, $I\left( z \right)$ is the second moment of area, E is the Young’s Modulus of materials to fabricate microneedle (2.79 GPa, according to Fig. 2d) and L is the length of the microneedle (~1.2 mm).

Considering the hollow pyramid shape of the needle, the $I\left( z \right)$ could be calculated as:

$$I\left( z \right)=\frac{{(D_{b}-\frac{z}{L}(D_{b}-D_{t})}^{4}-{(d_{b}-\frac{z}{L}(d_{b}-d_{t})}^{4}}{12}$$

$D_{t}$ is the tip outer length of the microneedle (~166 μm), $D_{b}$ is the base outer length of the needle base (~400 μm), $d_{t}$ is the tip inner length of the microneedle (~100 μm), $d_{b}$ is the base inner length of the needle base (~350 μm).

Therefore, the $F_{cr}$is calculated to be ~2.45 N, which is the critical value for the microneedle insertion into the skin to penetrate without breaking. In practice, the range of force used to place the microneedle into the skin by hand is usually less than its critical value to avoid microneedle breakage ^1,4,5^.


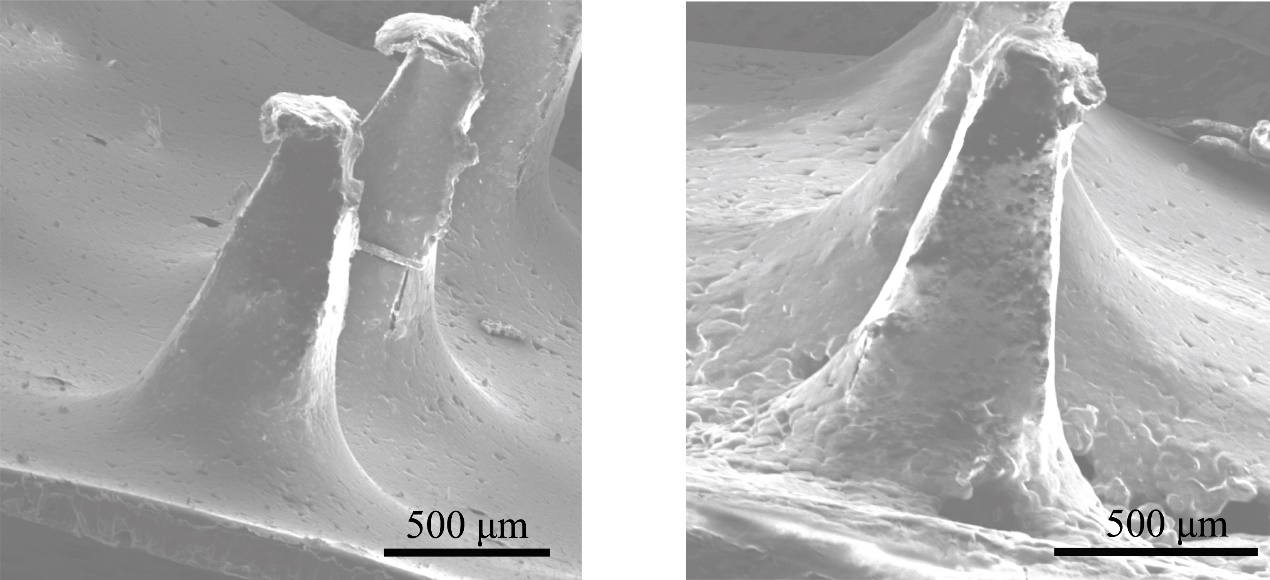


**Fig. S1** **SEM images of microneedles with different magnification.**


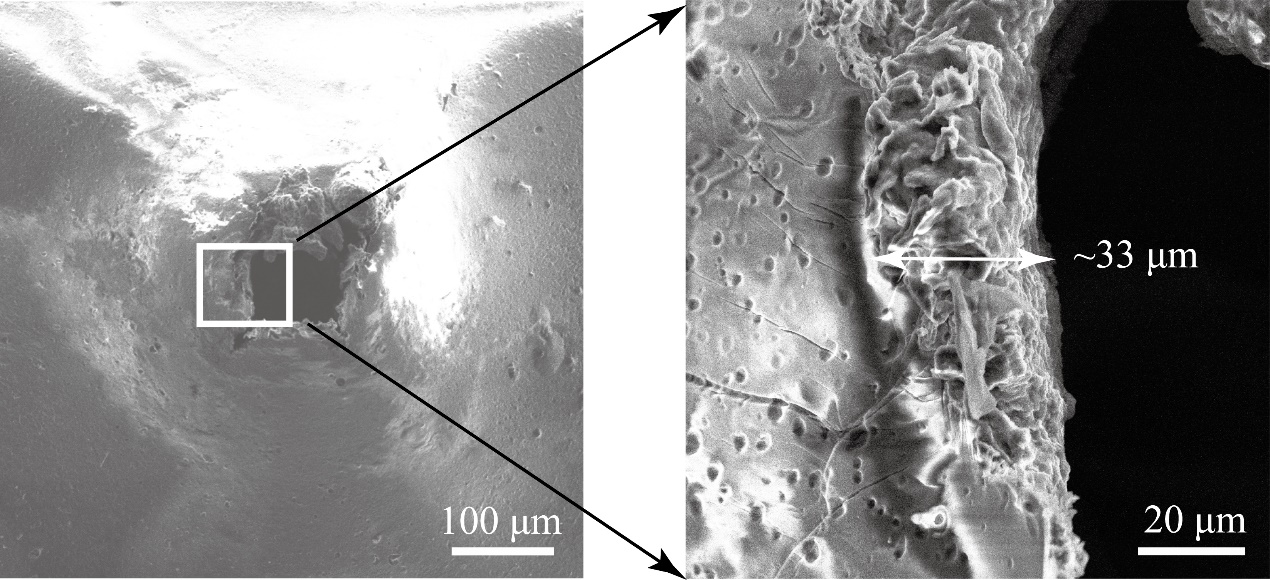


**Fig. S2** **SEM images of the microneedle’s tip hole.**


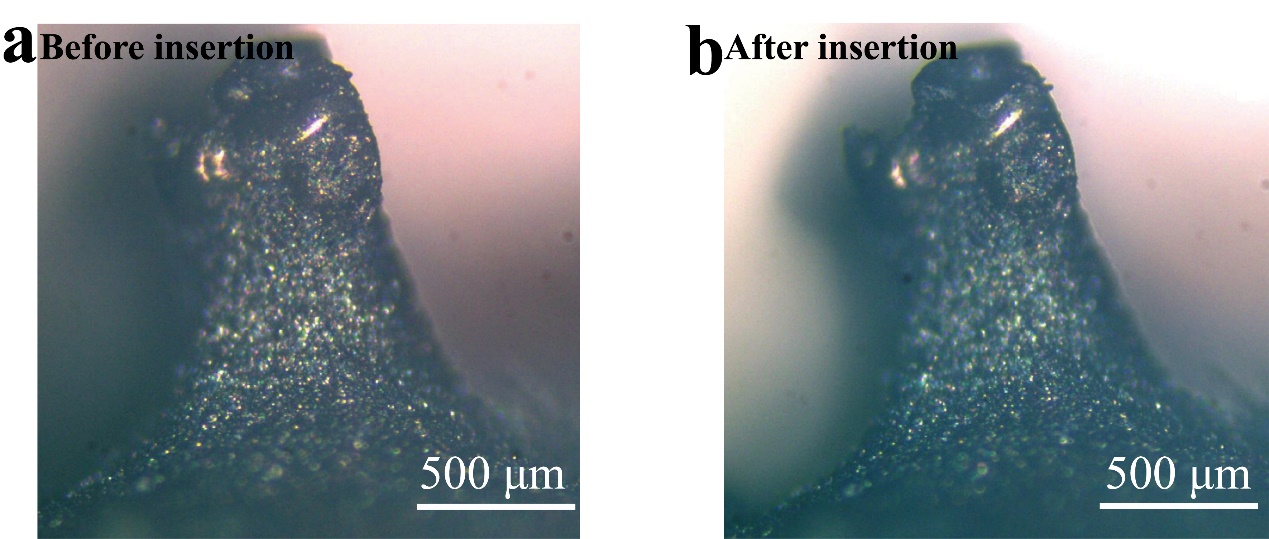


**Fig. S3 Optical images of the microneedle before and after insertion into the skin.** **a** Optical image of the microneedle before insertion into the skin. **b** Optical image of the microneedle after insertion into the skin. These images demonstrate that the microneedle could be successfully inserted into the skin without breaking.


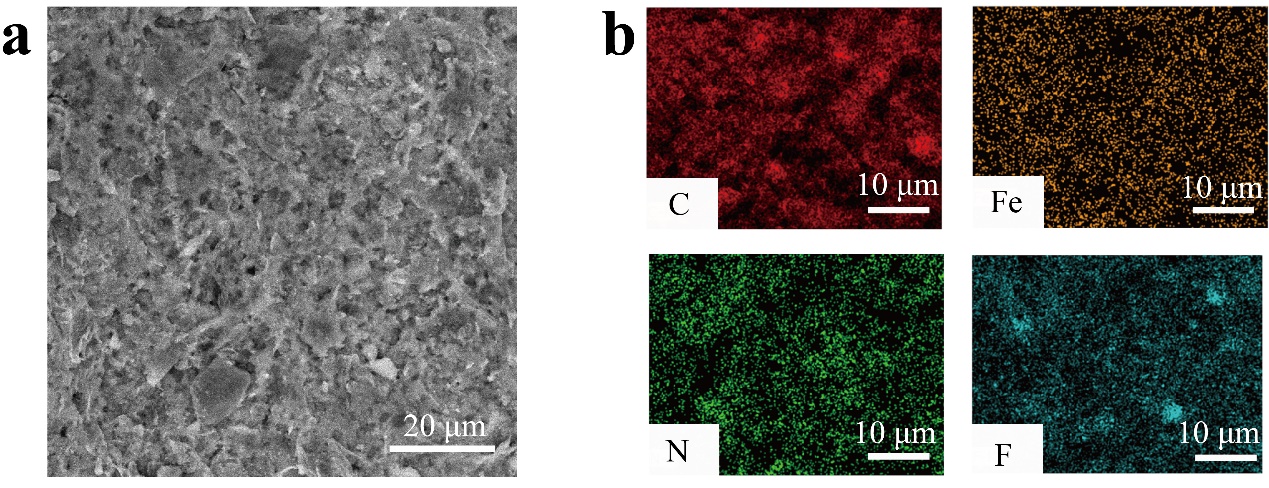


**Fig. S4** **SEM images and EDS analysis of the PS microneedle with graphene-PB electrode deposition.** **a** SEM image of the graphene-PB electrode on the microneedle. **b** Analysis of the graphene-PB electrode in EDS mapping.


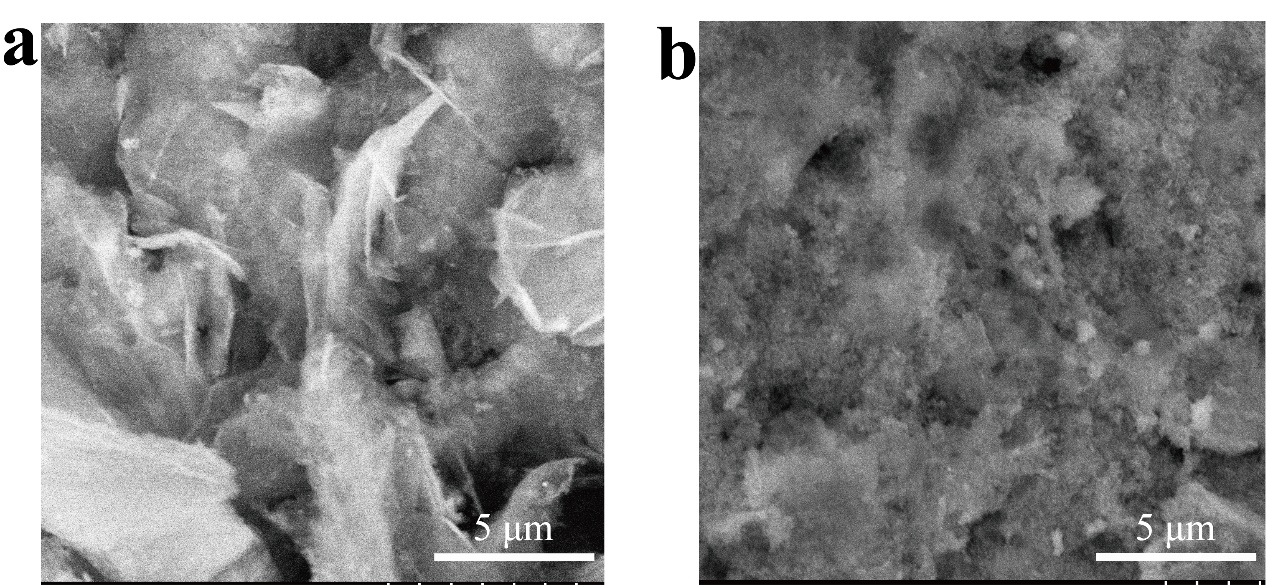


**Fig. S5** **SEM images of different graphene-PB electrodes.** **a** The PB to graphene ratio is 0 (wt%). **b** The PB to graphene ratio is 150%.


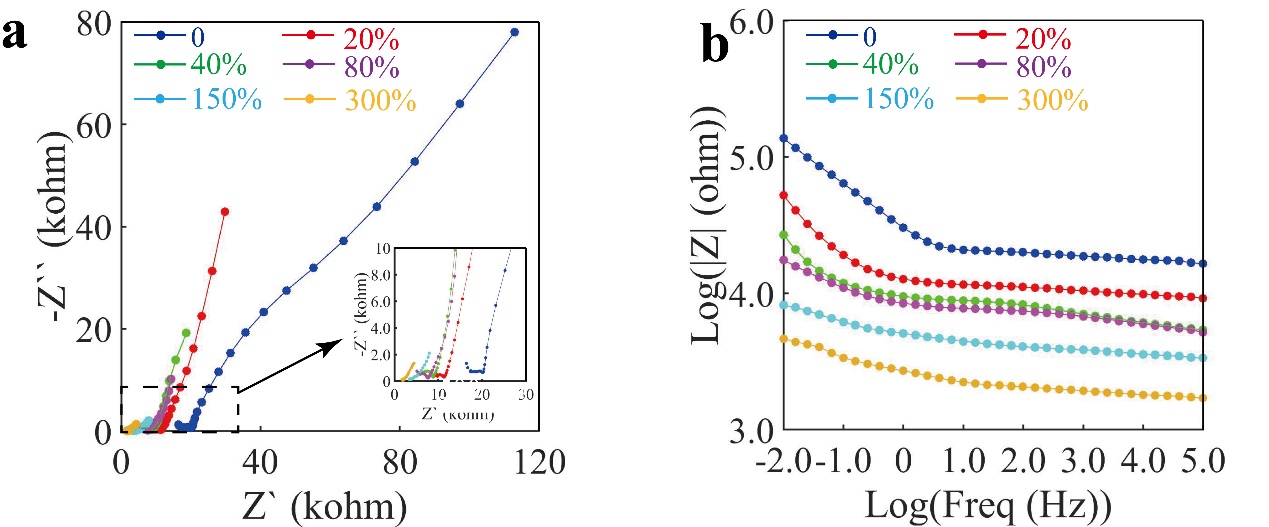


**Fig. S6** **Nyquist and Bode plots the microneedle biosensor in 0.1 M KCl/HCl with various PB: graphene ratios (wt%) in the scan frequency from 1 × 10^−2^ to 1 × 10^5^ Hz. a** Plot of Nyquist. **b** Plot of Bode.


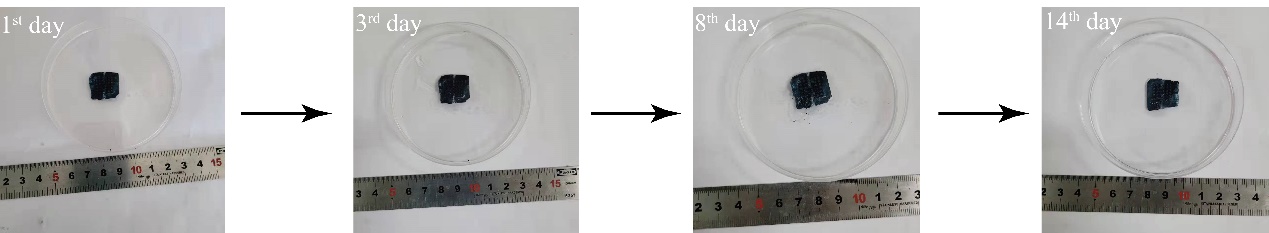


**Fig. S7** **Camera images of the PS microneedle with electrode deposition immersed into PBS (50 mM, pH 7.0) for 14 days.**


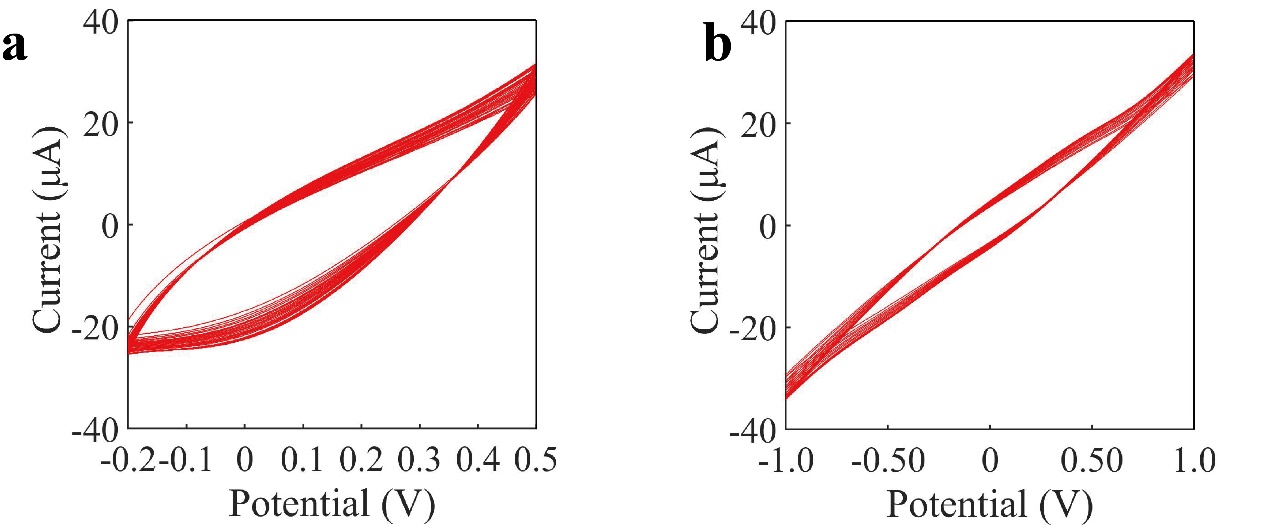


**Fig. S8** **Test of the graphene-PB electrode’s stability with CV measurements for 25 cycles.** **a** The sensor was in 0.1 M KCl/HCl (scan rate: 50 mV/s). **b** The sensor was PBS (50 mM, pH 7.0) containing 5 mM H_2_O_2_ (scan rate: 100 mV/s).


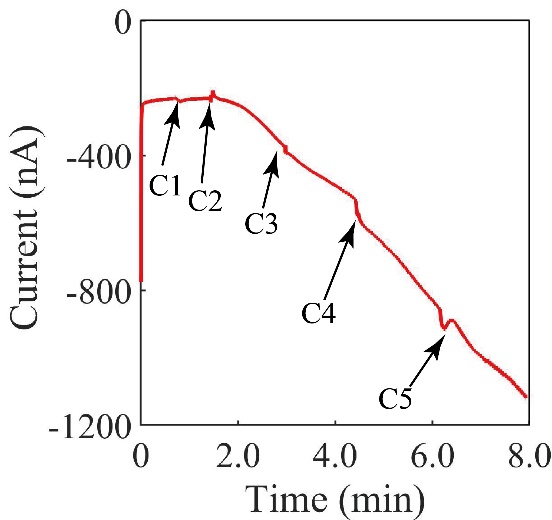


**Fig. S9** **The biosensor's current-versus-time response to subsequent additions of H_2_O_2_ in PBS (C1: 0.8 mM, C2: 2.2 mM, C3: 4.0 mM, C4: 5.0 mM, C5: 10 mM).**


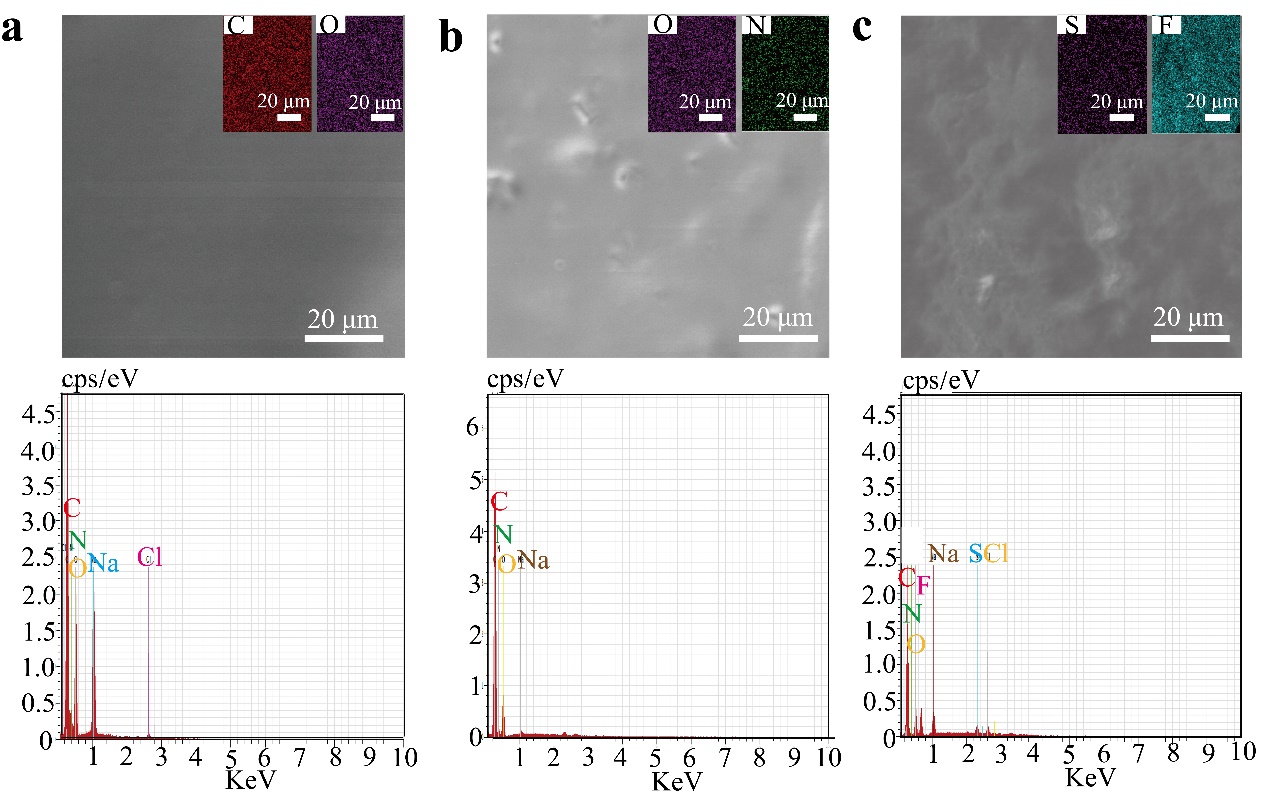


**Fig. S10** **The SEM images and EDS analyses of a graphene-PB electrode.** **a** After an enzyme layer deposition. **b** After a chitosan deposition. **c** After a Nafion membrane deposition.


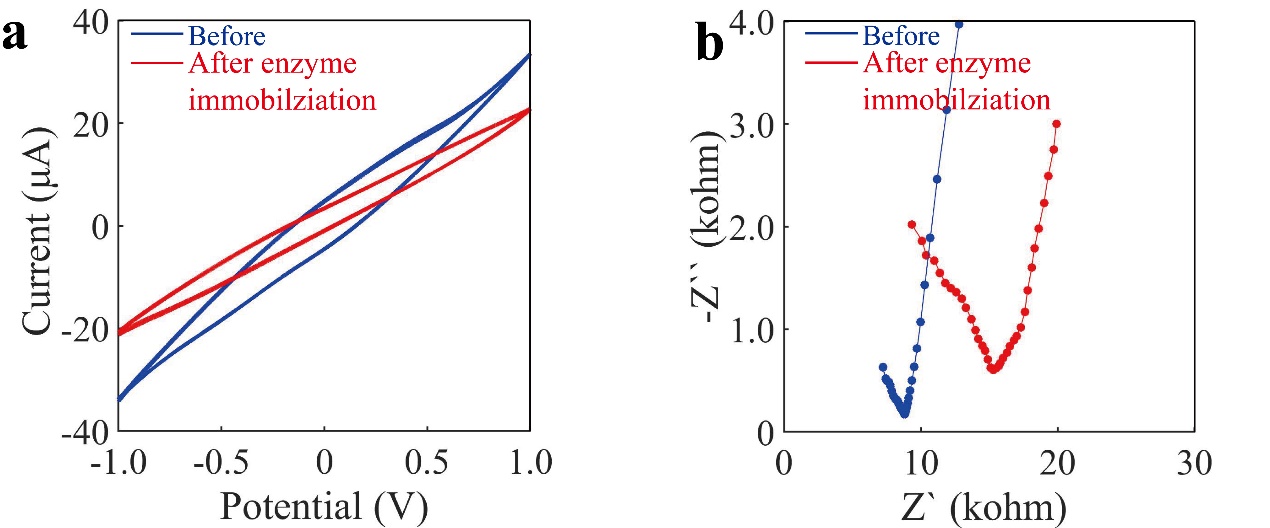


**Fig. S11** **The electrical characterization of the sensor in PBS (50 mM, pH 7.0) containing 5 mM H_2_O_2_ before and after the enzyme immobilization.** **a** The CV curves. **b** The EIS analysis.


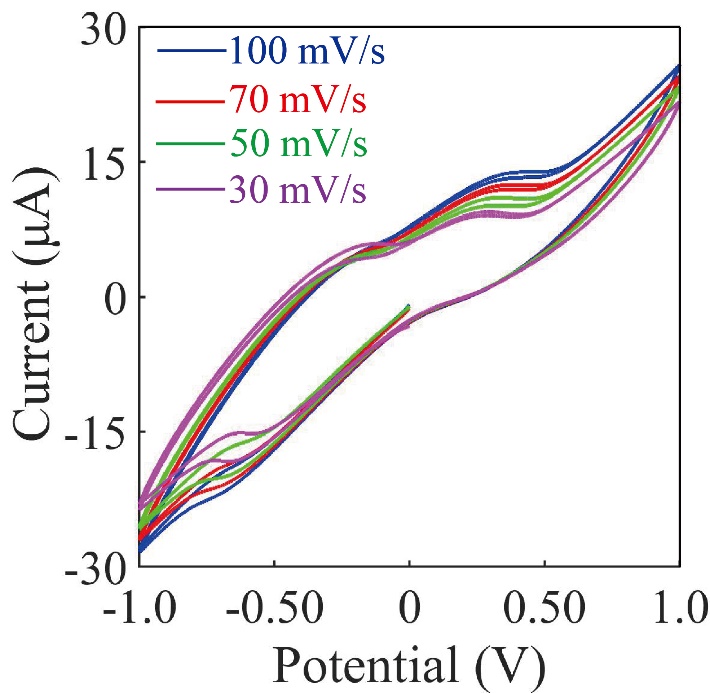


**Fig. S12** **The sensor's CV curves for detecting 5 mM glucose in PBS at various scan rates.**


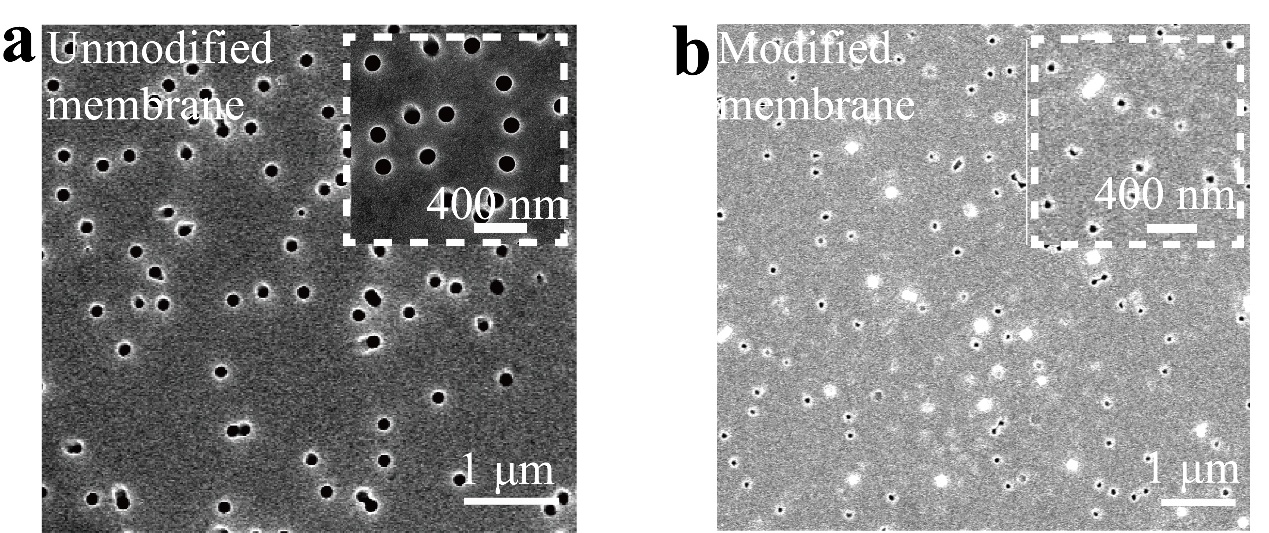


**Fig. S13** **The SEM images of the PC membranes.** **a** Unmodified membrane. **b** Modified membrane.


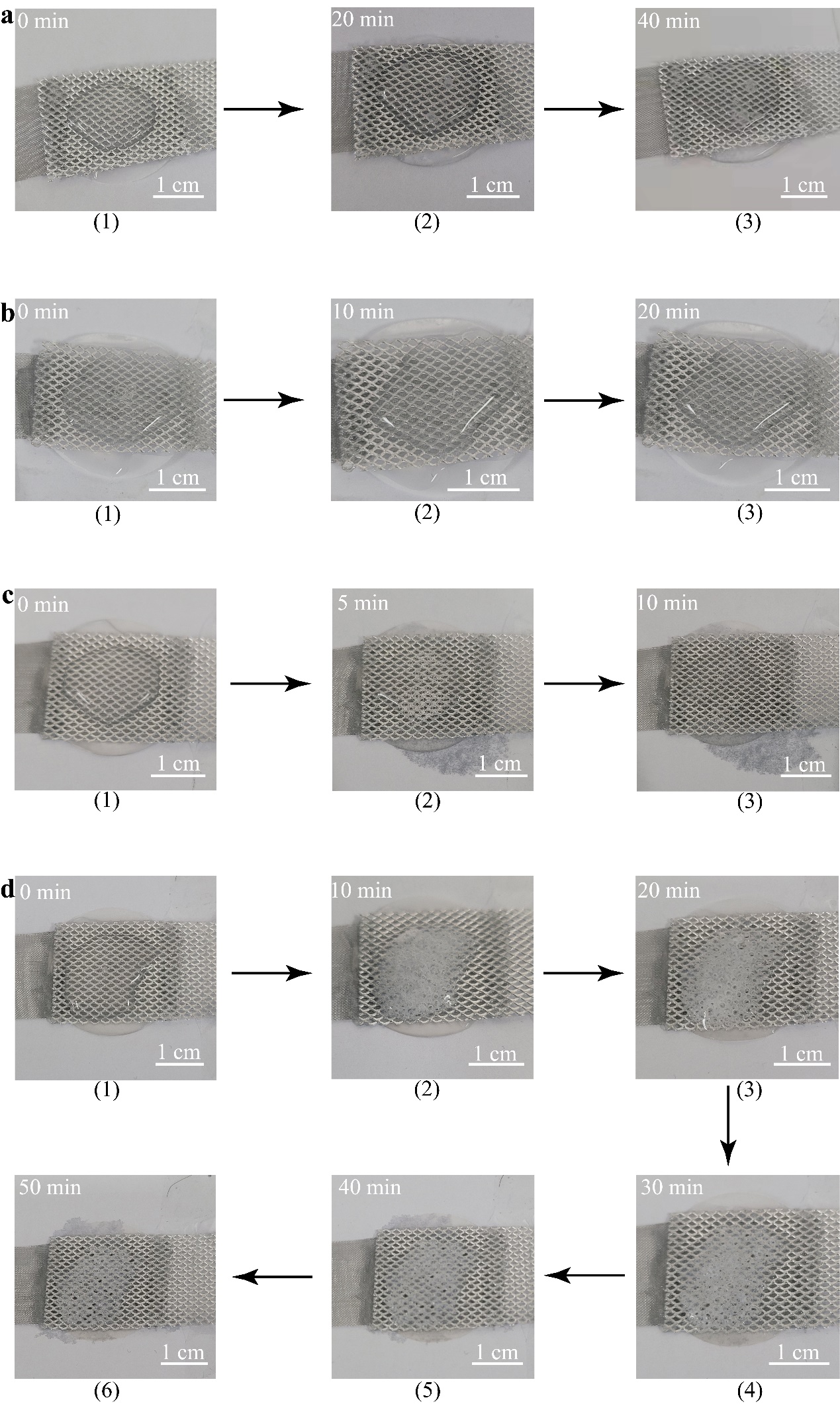


**Fig. S14** **The camera images of the electroosmotic micropump for the transportation of deionized water and insulin at 10 V, the micropump was placed on the paper.** **a** The unmodified membrane for releasing insulin (100 U/ml). The paper was not obviously got wet from (1)-(3), indicating that the insulin was almost not released. **b** The modified membrane for releasing deionized water without voltage application. The paper was obviously not got wet from (1)-(3), indicating that the deionized water could almost not be released without voltage application. **c** The modified membrane for releasing deionized water. The paper was obviously got wet from (1)-(3), indicating that the deionized water was released. **d** The modified membrane for releasing insulin (100 U/ml). The paper was gradually got wet from (1)-(6), indicating that the insulin was gradually released from the micropump.


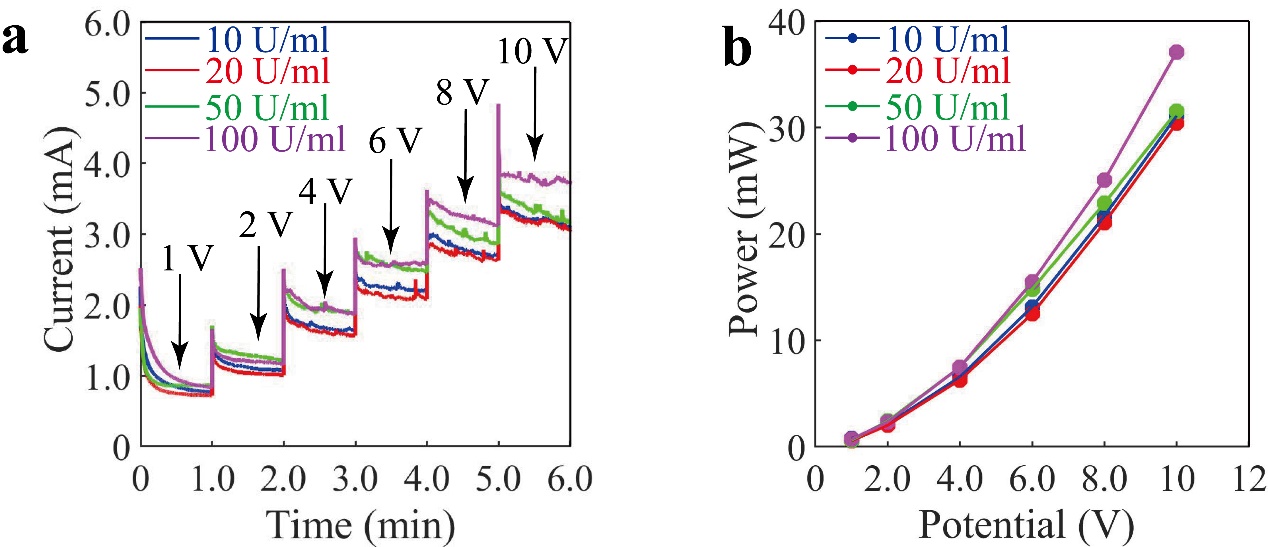


**Fig. S15** **The currents and the power consumptions of the micropump for releasing different insulin solutions.** **a** The current needed of the micropump under different potentials from 1 to 10 V for releasing different insulin solutions. **b** The power needed of the micropump under different potentials for releasing different insulin solutions.


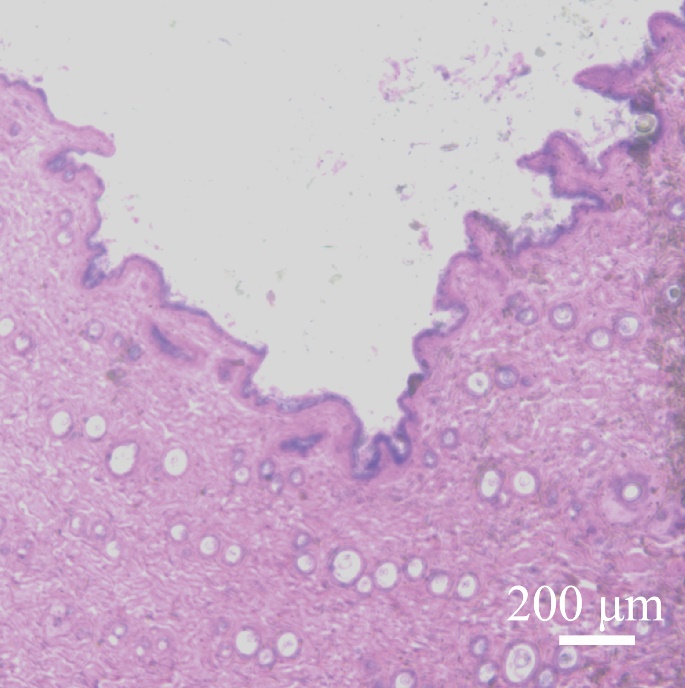


**Fig. S16** **The optical image of the** **hematoxylin and eosin-stained pierced rat’s back skin section after being applied with the microneedle.**


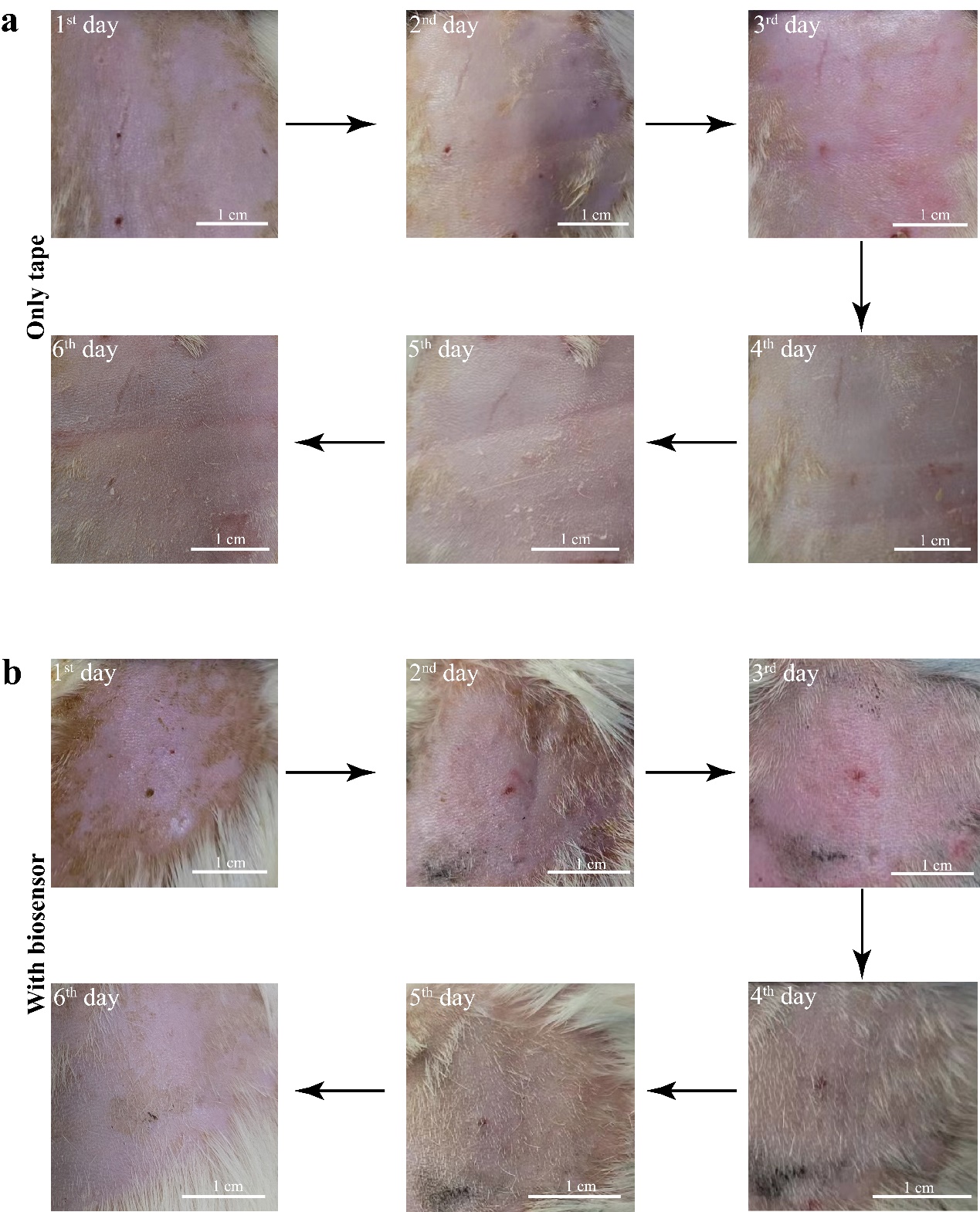


**Fig. S17 Camera images of the** **rats’ skin irritation test to evaluate the in-vivo biocompatibility of the biosensor. a** Only with the medical tape**; b** With the biosensor**.**


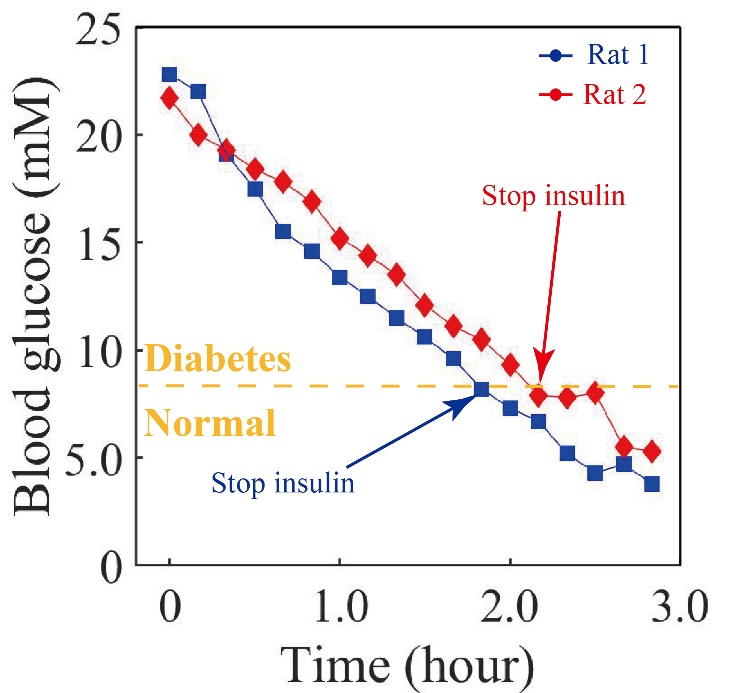


**Fig. S18** **The blood glucose versus time curves during the closed-loop management in another two rats without a glucose intake.**


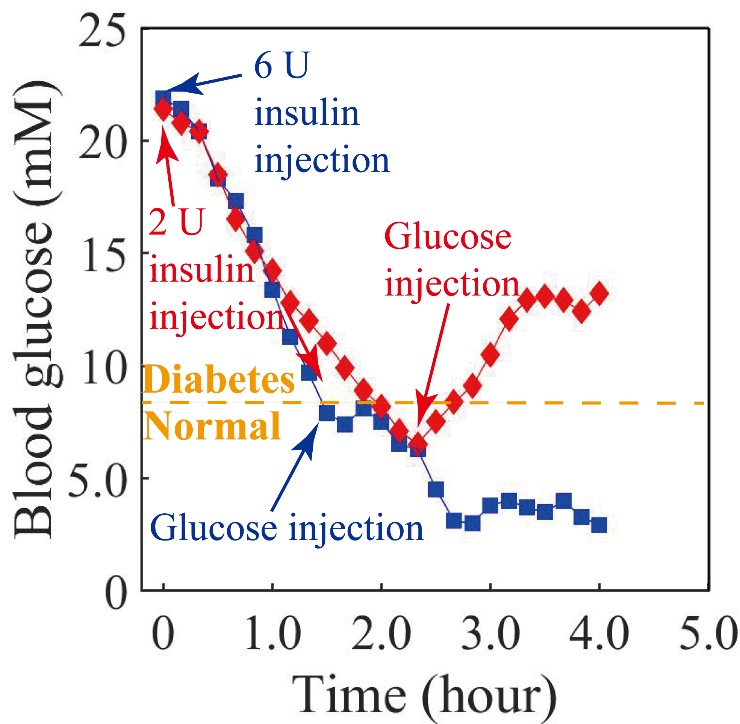


**Fig. S19 The blood glucose versus time curves with manual insulin injection in two rats.**


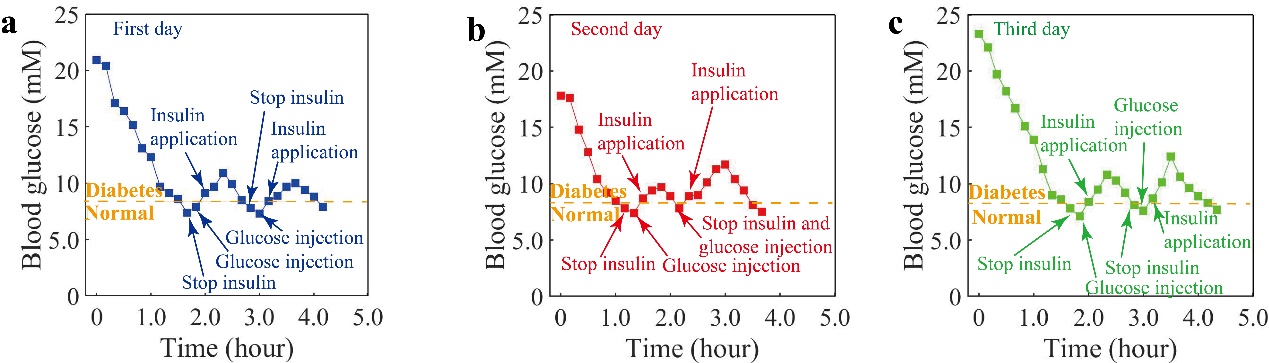


**Fig. S20 The blood glucose versus time curves during the closed-loop management with a glucose intake in one diabetic rat for three consecutive days. a** The first day. **b** The second day**. c** The third day.


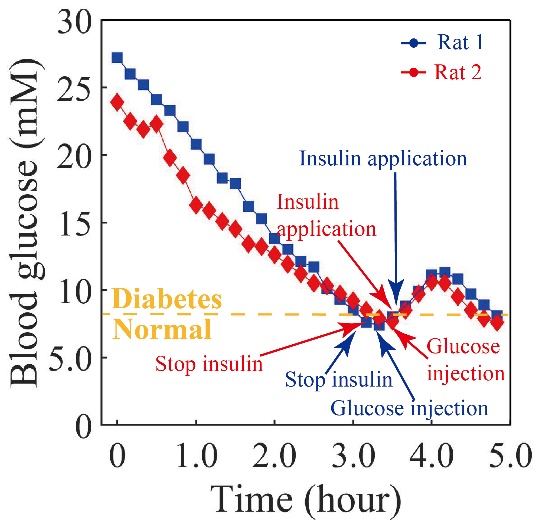


**Fig. S21** **The blood glucose versus time curves during the closed-loop management with a glucose intake in two terminal-stage diabetic rats (the modeling time was more than one month.**

**References**

1 Aggarwal, P. & Johnston, C. R. Geometrical effects in mechanical characterizing of microneedle for biomedical applications. *Sens. Actuat. B-Chem.* **102**, 226-234 (2004).

2 Smith, W. G. Analytic solutions for tapered column buckling. *Comput. Struct.* **28**, 677-681 (1988).

3 Le Thanh, H. *et al.* Low-Cost Fabrication of Hollow Microneedle Arrays Using CNC Machining and UV Lithography. *J. Microelectromech. Syst.* **24**, 1583-1593 (2015).

4 Zhang, Y., Jiang, G. H., Yu, W. J., Liu, D. P. & Xu, B. Microneedles fabricated from alginate and maltose for transdermal delivery of insulin on diabetic rats. *Mater. Sci. Eng. C-Mater. Biol. Appl.* **85**, 18-26 (2018).

5 Davis, S. P., Landis, B. J., Adams, Z. H., Allen, M. G. & Prausnitz, M. R. Insertion of microneedles into skin: measurement and prediction of insertion force and needle fracture force. *J. Biomech.* **37**, 1155-1163 (2004).
